# Evaluation of Topical Reconstituted HDL as a Treatment for Diabetic Wounds in Murine and Porcine Models

**DOI:** 10.3390/biom16071001

**Published:** 2026-07-09

**Authors:** Juan E. Camacho Londoño, Sharelle Sturgeon, Yun Dai, Alexey Navdaev, Padmapriya Ponnuswamy, Brandon H. Greene, Craig L. Duvall, Prarthana Patil, Joshua McCune, Mariah Bezold, Anil Dolgun, Justin R. Hamilton, Bronwyn A. Kingwell, Alberto B. Silva, Svetlana Didichenko, Helen Cao

**Affiliations:** 1 CSL Innovation GmbH, Emil-von-Behring-Straße 76, 5041 Marburg, Germany; 2 CSL Innovation, 655 Elizabeth Street, Melbourne, VIC 3000, Australiayun.dai@csl.com.au (Y.D.); helen.cao@csl.com.au (H.C.); 3 CSL Behring AG, Tellstrasse 18, 3014 Bern, Switzerlandalberto.silva@cslbehring.com (A.B.S.);; 4 Department of Biomedical Engineering, Vanderbilt University, Nashville, TN 37240, USA; craig.duvall@vanderbilt.edu (C.L.D.); joshua.t.mccune@vanderbilt.edu (J.M.); mariah.g.bezold@vanderbilt.edu (M.B.)

**Keywords:** high-density lipoprotein, diabetes, wound healing, porcine model

## Abstract

Chronic, non-healing foot ulcers are the most common complication of diabetes and are associated with high morbidity, mortality, and substantial health-care costs. Impaired wound healing in diabetes results from a complex pathophysiology involving persistent inflammation, interrupted re-epithelialization, impaired neovascularization, and defective granulation tissue formation. Reconstituted high-density lipoprotein (rHDL), with its antioxidant and anti-inflammatory properties, may counteract these processes and thereby promote wound repair. We investigated the effect of topical administration of rHDL in full thickness excisional wounds in murine and porcine diabetic models. rHDL significantly enhanced wound closure in diabetic mice in a dose-dependent manner as compared to phosphate-buffered saline (PBS) control. Histological and immunohisto-chemical analysis showed that rHDL-treated wounds had increased collagen deposition, a higher number of alpha-smooth muscle actin-positive cells, greater macrophage infiltration, and decreased neutrophil infiltration compared to PBS controls. In contrast, in the porcine model no wound healing improvement was observed after daily topical application of rHDL. Administration of rHDL enhances wound closure in a murine model of diabetic wound healing by promoting collagen production and modulating inflammation. However, lack of efficacy in a more physiologically relevant pre-clinical porcine model under the experimental conditions tested does not support further development of a topical rHDL formulation for diabetic wound healing indications.

## 1. Introduction

The global burden of diabetes is substantial and well documented, with an estimated 589 million adults living with diabetes worldwide, as reported in 2024 [1]. Diabetes is associated with an increased risk of mortality, cardiovascular disease, and a range of other health conditions [2,3]. Among its many complications, macrovascular (e.g., heart attack, stroke) and microvascular (e.g., kidney failure, neuropathy) sequelae are associated with long-term consequences that significantly impact patient quality of life [2,3]. One of the most severe and costly complications is diabetic foot ulcers (DFUs); these are the leading cause of non-traumatic lower limb amputation, and a significant cause of morbidity and mortality. It is estimated that up to one third of people with diabetes are likely to develop DFUs during their lifetime, requiring complex management approaches with high associated costs [4,5,6,7,8,9]. There have been several advancements in the management of ulcers, and the basic principles typically include offloading of plantar ulcers, debridement, revascularization when necessary, management of infection, and use of physiologic topical dressings. However, despite adherence to these principles, many ulcers will become chronic and fail to heal due to predisposing, precipitating, and perpetuating factors [10,11].

Impaired wound healing under diabetic conditions results from pathologic neurologic, vascular, or combined processes. Atherosclerosis is a common contributor to diabetes as it reduces blood flow to the extremities, which deprives tissues of essential oxygen and nutrients needed for wound healing [12,13]. Additionally, peripheral neuropathy is caused by prolonged hyperglycemia that damages both blood vessels and nerve tissue; this damage reduces sensation, particularly in the feet, making patients less likely to notice minor injuries or wounds. As a result, DFUs can develop into non-healing, chronic wounds, and their persistence is exacerbated by increased inflammation, recurrent infection, moisture-induced skin breakdown, and resulting hypoxia [6,12,13]. The pathophysiology behind impaired diabetic wound healing is complex, and over one hundred physiological factors have been shown to be impaired, including growth factor production, angiogenic response, macrophage function, collagen accumulation, epidermal barrier function, amount of granulation tissue, migration and proliferation of keratinocytes and fibroblasts, and epidermal innervation [14,15,16]. In summary, persistent inflammation prevents wounds from progressing toward successful healing.

Various interventions, including advanced wound dressings and topicals, physical energy-based therapies, regenerative scaffolds, cell-based therapies, and growth factor-based therapies, have been investigated and extensively reviewed. However, even some promising approaches, such as the use of growth factors, showed limited success [12,17,18,19,20,21]. One such example is becaplermin (Regranex^®^, a recombinant platelet-derived growth factor [PDGF]-BB), the only Food and Drug Administration-approved PDGF for the treatment of diabetic neuropathic ulcers since 1997, formulated to act as a first-line treatment after effective ulcer care [22,23].

Since chronic inflammation is a key element in the perpetuation of wounds, research efforts have focused on understanding the inflammatory mechanisms in order to identify new therapeutic targets. High-density lipoproteins (HDLs) have emerged as potential modulators of inflammation and tissue repair. The antioxidant and anti-inflammatory properties of HDLs related to their cholesterol efflux function have been well described and studied as therapeutic approaches for more than 2 decades, especially in the context of atherosclerosis [24,25,26,27,28]. The role of HDL particles in the regulation of tissue repair has been identified as a likely therapeutic target in the context of diabetic wound healing. Particularly, its role in inflammation, angiogenesis, vascular complications, macrophage and endothelial functions, among others, has been discussed [29,30,31,32]. The cholesterol-free apolipoprotein A-I (apoA-I)/phospholipid rHDL robustly drives ATP-binding cassette transporter A1 (ABCA1)-dependent cholesterol efflux and dampens inflammatory activation. These functional properties outperform those of native HDL and are potentially relevant to correcting the inflammatory environment of impaired wounds [26,33,34].

Furthermore, preclinical studies using murine models of wound healing, including diabetic models, have shown the beneficial effect of topical application of HDL when applied concomitantly with wound creation [35,36,37,38,39]. Genetic models further support this concept. Apolipoprotein E (apoE) deficiency delays wound healing, while combined apoE/apoA-I deficiency causes severe skin and inflammatory alterations, underscoring the relevance of HDL pathways in cutaneous repair [35,40].

In this study, we evaluated the hypothesis that reconstituted (rHDL) may exert beneficial effects when applied in a therapeutic setting (specifically 2 days post-wound creation) in a diabetic murine wound model. We also extended the investigation to a translational porcine model to assess the efficacy in a system more representative of human skin physiology.

## 2. Materials and Methods

### 2.1. Preparation of rHDL

rHDL particles containing 2 molecules of apolipoprotein A-I (apoA-I) and 132 molecules of phosphatidylcholine (PC) per particle (apoA-I to PC molar ratio 1:66), with a predominant rHDL species displaying a hydrodynamic diameter of approximately 7.9 nm, were prepared as previously described [26].

### 2.2. Protein Labeling

Labeling of rHDL and albumin with Sulfo-Cy5.5 NHS ester (Lumiprobe, Germany) was performed according to the manufacturer’s recommendations with minimal modifications. Briefly, rHDL or albumin (both at 10 mg protein/mL) solubilized in phosphate-buffered saline (PBS) (Gibco, Thermo Fisher Scientific, Reinach, Switzerland) were mixed with sulfo-Cy5.5 NHS ester (10 mg/mL) dissolved in water at a 3-fold molar excess of sulfo-Cy5.5 NHS ester. Labelling was carried out for 30 min at 4 °C. The reaction was terminated by adding 1 M glycine (Sigma-Aldrich, Merck, Darmstadt, Germany) (pH 7.5) up to a final concentration of 100 mM. Unbound dye was removed by ultrafiltration on Amicon Ultra Centrifugal Filters (Ultracel 50 K; Merck Millipore, Darmstadt, Germany). Degree of labeling with Cy5.5 was calculated according to the manufacturer’s recommendations. The labeling procedures yielded one to two Cy5.5 molecules per apoA-I molecule in HDL particles. A similar degree of labelling was found for albumin.

### 2.3. Preparation of rHDL Formulations Containing 20% (Weight/Volume [w/v]) Pluronic F-127

Pluronic F-127 is an FDA-approved hydrogel that is temperature-sensitive, injectable, biodegradable, non-toxic, and biocompatible. A 20% (*w/v*) Pluronic F-127 solution behaves as a thermosensitive “smart” polymer, remaining in liquid form at low temperatures (e.g., 4 °C) while rapidly undergoing sol–gel transition at physiological temperatures. As a result of these properties, Pluronic F-127 formulations are widely used for drug delivery, wound healing, and tissue engineering applications [41].

A total of 700 mL of sterile PBS (Gibco) was added to a glass bottle and cooled to 4 °C. Pluronic F 127 powder (Sigma Aldrich) was gradually added to the cold PBS to reach 30% (*w/v*) and gently mixed overnight at 4 °C using a magnetic stirrer to ensure complete dissolution. The final volume was adjusted to 1000 mL with PBS. The solution was sterilized by autoclaving and stored at 4 °C until further use.

rHDL (30 mg protein/mL) was diluted in PBS and mixed with 30% (*w/v*) Pluronic F-127 solution on ice to obtain the formulations, containing rHDL at concentrations from 1 to 10 mg protein/mL and 20% (*w/v*) Pluronic F-127. rHDL formulations were immediately aliquoted into sterile cryogenic tubes and snap-frozen in liquid nitrogen. The frozen aliquots were stored at −70 °C until use. During application, the formulations were maintained at a low temperature to preserve liquid handling properties before rapid gelation upon contact with the wound surface.

### 2.4. Non-Denaturing Polyacrylamide Gradient Gel Electrophoresis

Non-denaturing polyacrylamide gradient gel electrophoresis was performed as previously described [34]. Gels were stained using the GelCode Blue Stain Reagent (Thermo Scientific, Reinach, Switzerland) according to the manufacturer’s recommendations.

### 2.5. In Vitro Uptake Assay

Murine macrophage cell line RAW264.7 was used to measure the uptake of fluorescently labeled rHDL (Cy5.5-rHDL) encapsulated in 20% Pluronic F-127 gel. Briefly, RAW264.7 cells (ATCC) were seeded into 12-well chamber slides at a density of 0.25 × 10^6^ cells/mL (250 µL per well) in Dulbecco’s Modified Eagle Medium (DMEM) supplemented with 10% fetal calf serum (FCS) and 100 units/mL penicillin and 100 μg/mL streptomycin (all reagents from Gibco). After 24 h, the medium was removed, and a cold DMEM containing either Cy5.5-rHDL or Cy5.5-rHDL/20% Pluronic F-127 or Cy5.5-Albumin (all compounds were at 1 mg protein/mL) was added to the cells. Cells were then incubated at 37 °C for 30 min or 2 h in a humidified atmosphere with 5% CO_2_. Following incubation, cells were washed with cold PBS to remove unbound compounds. Cell-associated Cy5.5-rHDL or Cy5.5-Albumin was visualized using fluorescence microscopy.

### 2.6. Stimulation of Peripheral Blood Mononuclear Cells (PBMCs) and IL-8 Measurements

PBMCs were isolated from buffy coats as previously described [33]. PBMCs were cultured overnight in RPMI 1640 supplemented with 10% (*v*/*v*) FCS, 2 mM L glutamine, 100 units/mL penicillin, and 100 μg/mL streptomycin (all reagents were from Gibco) at a density of 1.5 × 10^6^ cells per well in 48-well plates. The cells were then stimulated with lipopolysaccharide (LPS, 100 ng/mL final concentration) in the presence or absence of rHDL or rHDL/20% Pluronic F-127 formulation (both at 1 mg protein/mL). Following 6 h of incubation, interleukin-8 (IL-8) levels were measured in cell culture supernatants using the Human IL-8/CXCL8 enzyme-linked immunosorbent assay (ELISA) kit (R&D Systems, Zug, Switzerland).

### 2.7. Animal Experiments

#### 2.7.1. Ethics Statement

The study protocol for mouse studies was approved by the CSL Animal Ethics Committee (#2011, July 2020). The pilot swine study was approved and performed according to the Vanderbilt University Institutional Animal Care and Use Committee (IACUC), USA (M1700076, May 2022). The pig wound healing studies were sponsored by CSL Behring and conducted in collaboration with Vanderbilt University and Altasciences Preclinical, Columbia, USA. The Testing Facility is accredited by the Association for Assessment and Accreditation of Laboratory Animal Care (AAALAC), has an Animal Welfare Assurance with the Office of Laboratory Animal Welfare (OLAW), is registered with the United States Department of Agriculture (USDA), and has an Institutional Animal Care and Use Committee (IACUC) responsible for the testing facility’s compliance with applicable laws and regulations concerning the humane care and use of laboratory animals. All animal procedures were conducted in accordance with the ethical standards and guidelines for the care and use of laboratory animals. All efforts were made to minimize animal suffering and to reduce the number of animals used.

#### 2.7.2. Test Species and Animal Husbandry

C57BL/6J mice (n = 132) were purchased from the Animal Resources Centre, Australia. They were acclimatized to their environment for at least 1 week before the procedures and were allowed food and water *ad libitum*. Five animals per cage were housed before surgery and alone post-procedure in a temperature-controlled animal facility with a 12 h light/dark cycle.

In the pilot swine study, Yorkshire female pigs were purchased from Oakhill Genetics. Animals weighing 27–36 kgs and 16–18 weeks of age were used for the experiment. The animals were acclimatized for 3–5 days before surgery, with food and water provided *ad libitum*. Animals were fasted for 12–18 h prior to surgical procedures and dressing changes. Animals were treated sequentially; the second animal was treated 1 month after the first one.

The studies in diabetic pigs were performed at Altasciences Preclinical, Columbia, USA. Animals were housed in a facility provided with stainless steel, self-spanned polyvinyl chloride (PVC)-coated expanded metal flooring and kept at an appropriate temperature based on the animal’s age (>6 months: 61–81 °F [16–27 °C]) under a 12 h light/dark cycle, except during designated procedures. Species-specific enrichment was included and drinking water was provided *ad libitum* via an on-site filtered, deep-well water source routinely analyzed for contaminants. The diet (non-certified Purina S-9 swine diet) was provided based on the animal’s age and usually in 2 feeds per day. Veterinary care was available throughout the course of the study and animals were examined by veterinary staff as warranted by clinical signs. Eight naïve diabetic (induced by alloxan administration to achieve fasting blood glucose levels of 150 mg/dL or above), castrated, male, catheterized Yucatan miniature pigs were used in this study. Hyperglycemia was regulated by insulin (Humalog and Lantus, or equivalent) based on the glucose levels of each animal and at the discretion of trained veterinary staff. Efforts were made to maintain an acceptable blood glucose level (reference target range: 150–500 mg/dL, measured at least once daily) for the specific animal during the study period. Insulin and/or feed amounts were adjusted as needed to maintain acceptable blood glucose levels; in isolated cases of hypoglycemia, oral administration of 50% dextrose and/or food offerings (e.g., liquid nutrition, canned food, treats, etc.), intravenous (i.v.) administration of 5–50% dextrose, or other treatments as recommended by Altasciences veterinary staff were considered. To support model characterization, comprehensive clinical pathology data were collected pre-wounding and on day 18 and are summarized in Table S1. Few parameters showed minor deviations in some values from historical miniature swine reference ranges (e.g., lower aspartate aminotransferase, calcium, total protein, inorganic phosphorus; higher cholesterol) [42]; however, these changes were not accompanied by clinical signs of dehydration, metabolic disturbance, or neurologic impairment, and no animals exhibited findings consistent with diabetic ketoacidosis or hyperosmolar hyperglycemic state during the study (except for one isolated vomiting event). Wounding surgery was performed in animals after 2 months of confirmed alloxan-induced diabetes.

#### 2.7.3. Studies in Diabetic Mice

Diabetes was induced in 8-week-old male C57BL/6J mice (20–23 g, body weight) via a single intraperitoneal (i.p.) administration of streptozotocin (Sigma) at 165 mg/kg 2 weeks prior to the surgery. Blood glucose levels were measured using the Accu-CHEK Performa Blood Glucometer and glucose levels of 15.0 mmol/L (270 mg/dL) or above were considered diabetic; in addition, body weight was monitored as an important wellbeing indicator of the diabetic animals in the wound healing studies (Figure S1A–C). Randomization was stratified by body weight, cage, and glucose levels to allocate mice across the different treatment groups. Animals were housed under identical conditions, and experimental procedures (e.g., anesthesia, wound size measurement, and rHDL/control administration) were staggered to ensure comparable treatment duration across all animals. All histological and immunohistochemical analyses were conducted in a blinded manner.

The wound healing model was conducted as previously described [43] and additional details are reported in the Supplementary Materials. A total of 20 µL of rHDL in PBS or PBS alone was topically applied (either as a liquid formulation or gel formulation in 20% Pluronic F-127 in PBS) to the wounds daily during the study course after wound surgery. Wound sizes were measured daily along the X, Y, and Z axes using calipers. At the end of the study, the mice were humanely killed, and wound tissues were excised and processed for histological and immunohistochemical analysis. Collagen deposition was assessed by Masson’s trichrome staining, while immunohistochemical analyses included detection of neutrophils (Ly6G/Ly6C), macrophages (CD68), and αSMA-positive cells. Detailed histological procedures, antibody information, staining conditions, imaging acquisition, and quantification methods are provided in the Supplementary Materials, including the Major Resources Table.

#### 2.7.4. Pilot Studies in Pigs

Two adolescent female Yorkshire pigs were anesthetized (see Supplementary Materials) and under sterile conditions, full-thickness excisional wounds (each with a total surface area of 2 cm^2^) were then created within two rows of nine (18 wounds in total), each positioned approximately 3 cm from the spine. All treatment groups were randomized across different positions along the dorsal region of the animals to account for positional anatomical variability inherent to this model. All measurements and analyses were performed by personnel blinded to treatment allocation. Wounds were treated on post-operative days (POD) 1, 3, 6, 8, and 10. Excede^®^ (Zoetis) 5 mg/kg was delivered intramuscularly (i.m.) as a systemic antibiotic on the day of surgery and 7 days post-surgery. Wounds were monitored and routinely evaluated by veterinary staff.

Wound size was calculated as a percentage of wound area measured on post-operative day (POD) 0 using ImageJ 1.53 software [32]. Tissue samples were fixed in 10% neutral buffered formalin for 48 h and were then dehydrated in a graded ethanol series, exposed to xylene, and embedded in paraffin. Tissue sections (5 µm thick) were deparaffinized in gradients of xylene and ethanol and rehydrated in Tris-Buffered Saline/0.1% Tween 20 (TBST) buffer. Gomori’s Trichrome and hematoxylin and eosin (H&E) stains were performed according to the manufacturer’s recommendations. Additional details are provided in Supplementary Materials.

#### 2.7.5. Studies in Diabetic Yucatan Miniature Pigs

After approximately 2 months of confirmed diabetes induction (Figure S1E) and at an age between 8 and 12 months (25–50 kg), eight male animals were anesthetized (see Supplementary Materials) to induce wounds. The wound sites were positioned along a paraspinal column on each side, ensuring the column remained between the crest of the shoulders and the ilium. Each animal had eight wound sites (one row of four sites per side) with a diameter of 2.5 cm (~5 cm^2^), and appropriate depth (full thickness) spaced at least 3 cm apart (Figure S2). Clinical blood parameters were monitored during the acclimation (prior to randomization) and prior to termination (Table S1).

#### 2.7.6. Dose Administration, Dermal Scoring, and Wound Planimetry in Diabetic Pigs

Sample size calculations were based on data from previous studies, including results from the pilot study. Assuming a normal distribution, estimates of within-subject and between-subject variance components for the primary endpoint, wound closure (%), were determined using a linear mixed-effects (LME) regression model fitted to data from internal preliminary experiments. The resulting estimates of SD = 15.3% (within-subject) and SD = 11.3% (between-subject) were subsequently used for power analyses using LME regression models across different group sizes and effect sizes, expressed as mean differences (MD) in wound closure between groups at the end-of-study time point. These analyses indicated a benchmark statistical power > 80% with a group size of N = 8 animals for effect sizes corresponding to MD ≥ 20%. The calculations also accounted for the correction of two-sided *p*-values for multiple comparisons between the k = 3 active treatment dose groups and each control group. Animals were randomly assigned to one of the eight treatment schemes. Treatment schemes within animals were balanced to ensure that all treatments were applied across all possible anatomical wound positions (see Supplemental Methods). Operators and personnel involved in data collection and sample analysis (e.g., histopathology) were unaware of the treatments and their allocation. This information was only disclosed after completion of the analyses, when required for comparison of treatment effects. Vehicle (20% Pluronic F-127 in PBS), rHDL in 20% Pluronic F-127 in PBS gel formulation (2, 5 and 10 mg/mL), or albumin control (in 20% Pluronic F-127 gel in PBS; protein-matched to the highest rHDL concentration) were applied directly to the designated wound site(s) daily, from dosing phase Day 1 for up to 18 days (or when ~70–80% wound closure was achieved in any of the groups with the fastest healing). Concentrations higher than 10 mg/mL rHDL could not be achieved in the gel formulation under the experimental conditions used in this study. An appropriate sterile pipette tip was used to add 0.5 mL of the formulation directly to the middle of the designated wound bed to cover it completely (without spreading over), and allowed to solidify (~1–2 min). Becaplermin (Regranex^®^, Smith & Nephew) was applied at 0.3 g per wound, corresponding to approximately 1.2 cm from a 15 g tube, as recommended by the manufacturer and calculated based on the initial wound size (4.9 cm^2^). According to the manufacturer’s dosing instructions, each square centimeter of wound surface requires approximately 0.25 cm of gel from a 15 g tube. For example, a wound measuring 4 cm × 2 cm would require approximately 2 cm of gel [(4 × 2) ÷ 4 = 2]. Becaplermin was administered once daily in the present study, whereas clinical administration in patients is typically performed twice daily (every 12 h). The once-daily application schedule was selected due to technical and logistical constraints associated with the experimental protocol. Each animal received each treatment in at least one wound (Figure S2). Each wound site was then covered with a barrier dressing of non-adherent sterile gauze and transparent film. Once all sites were dosed and barrier dressing applied, the entire wound area was covered with a layer of foam pad and tear-resistant mesh (or stockinette) to prevent dressing materials from moving. Wounds were scored using a modified Bates-Jensen dermal scoring prior to dosing on Days 1, 5, 8, 12, 14, 17, and 18 of the dosing phase, and features such as wound edge, exudate type and amount, granulation tissue, and epithelialization were considered. On the same days, planimetry photography was performed prior to dose application using a SilhouetteStar ARANZ Medical camera (see Supplementary Materials). On Day 18, a complete gross necropsy was performed on all study animals, and the wound sites were excised with a 0.2 cm margin of surrounding normal skin (peri-wound area, Figure S2). The wound sites were cut in half and the halves that contained peri-wound areas (Figure S2) were fixed in 10% neutral buffered formalin and processed to obtain paraffin blocks and histological slides at Altasciences Preclinical Columbia, LLC, 562 State Road DD, Auxvasse, MO 65231, USA, for histopathological evaluation at StageBio, USA. Blocks were further shipped to Anapath Services GmbH, Switzerland for a second independent pathologist analysis.

#### 2.7.7. Histopathological Analysis of Samples from Diabetic Pigs

Two independent pathologists (at StageBio and Anapath), analyzed tissue sections from each wound site. Histomorphometry by Image Analysis was done at Anapath. The sections were stained with H&E to assess general morphology, and Mason’s Trichrome and Herovici stainings were used to assess collagen maturity. The slides were evaluated according to the International Organization for Standardization (ISO) 10993-6:2016, Annex E standard [44], with additional assessment of wound-bed specific features. The slides were evaluated for inflammation and inflammatory cell types, wound bed healing, tissue response, and reactivity score (ISO 10993-6:2016). Additional tissue sections were generated and analyzed at AnaPath, using histomorphometry by image analysis and scoring defined by ISO 10993-6:2016. The main histopathological wound healing parameters are summarized in Table 1 and extended in the Supplementary Materials in Tables S3 and S4. Diagnostic criteria and terminology used throughout the study were based on recognized texts, current scientific literature, as well as Registry of Industrial Toxicology Animal-data (RITA), Society of Toxicologic Pathology (STP), and the International Harmonization of Nomenclature and Diagnostic (INHAND) terminology. Inflammatory cell populations, including polymorphonuclear cells, lymphocytes, plasma cells, macrophages, and giant cells, were identified morphologically by experienced pathologists according to established histopathological criteria routinely used in diagnostic and toxicologic pathology.

### 2.8. Statistical Planning and Analysis

Distinct statistical approaches were applied to mouse and pig studies to reflect differences in design and endpoints.

#### 2.8.1. Analysis of Studies in Diabetic Mice

For the therapeutic treatment study, i.e., rHDL and PBS administration commenced 2 days after wound surgery, data were expressed as mean ± standard deviation (SD) of the percentage wound closure. Differences between PBS and treatment groups were analyzed using a linear mixed-effects model. The model included mouse as a random effect to account for correlation between multiple wounds within the same animal and repeated measurements over time. Dunnett’s multiple comparison test was applied to assess differences between rHDL and PBS.

For the prophylactic treatment study, rHDL and PBS administration commenced on the same day as wound surgery, and data were expressed as mean ± SD of the percentage wound closure as described above. Differences between PBS and rHDL treatment were analyzed using a linear mixed-effects model with between-subject treatment effects. The model included mouse as a random effect to account for correlation between multiple wounds within the same animal and repeated measurements over time. For the histological and immunohistochemical analysis of wounds, the nonparametric Mann–Whitney U test was used to compare the percentage of αSMA-positive area, CD68-positive area, collagen-positive area, and neutrophil-positive area between the rHDL and vehicle groups. The group sizes were informed by historical data and practical experience rather than formal sample size calculations. For the murine studies, the mouse was considered the experimental unit. Statistical significance was set as *p* < 0.05.

#### 2.8.2. Analysis of Studies in Diabetic Pigs

Wound area closure responses were analyzed using a generalized least squares (GLS) model fit for the longitudinal data [45] to account for the correlation within repeated measures on the same subject across multiple time points. The model included terms adjusting for wound site position (cephalic/caudal). Comparisons were calculated as mean differences (MD) between treatment groups on Day 18. For the porcine studies, the wound was considered the experimental unit. Comparisons of sites treated with rHDL versus vehicle, becaplermin, and albumin, respectively, were grouped into families, and *p*-values and 95% confidence intervals (CI) within each family were adjusted for multiple testing using the single step method of Hothorn, Bretz, and Westfall (2008) [46].

The effect of treatment on discrete, ordinal scoring outcomes (Bates-Jensen, histopathology, and histopathology scores) on Day 18 was analyzed by pairwise comparisons using the nonparametric Hodges-Lehmann aligned ranks test [47] with individual animals as the blocking factor. For the total score, the 95% CI for the Hodges–Lehmann estimator of treatment effect was calculated by using the shift model and inverting the rank test as described by Bauer (1972) [48]. For families of comparisons as described above, *p*-values and 95% CI within each family were adjusted for multiple testing using the Bonferroni–Holm method. Mean values from the histomorphometry by image analysis were compared using ordinary one-way ANOVA and *post hoc* Tukey’s multiple comparisons test with adjusted *p*-value.

## 3. Results

### 3.1. Wound Healing in Diabetic Mice

Topical administration of rHDL enhances wound healing in diabetic mice under therapeutic conditions and in a dose-dependent manner.

**Figure 1:**
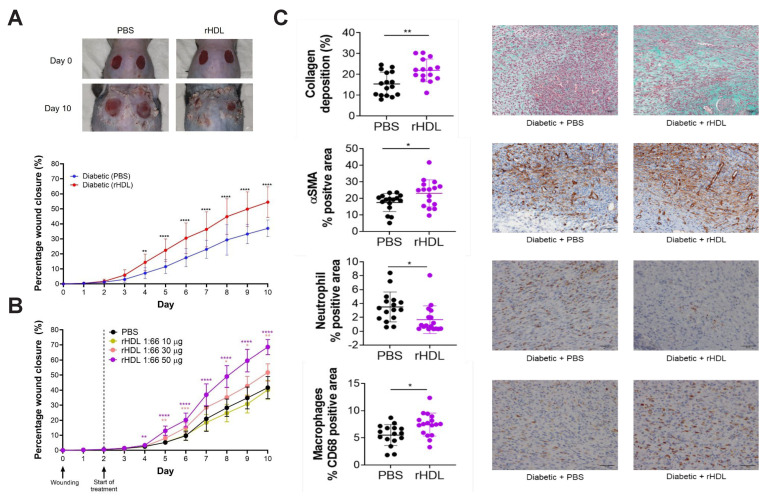
Topical administration of rHDL enhanced wound healing in diabetic mice under therapeutic conditions. (**A**) Wound closure was analyzed in diabetic C57BL/6J mice (PBS: n = 12 mice; rHDL: n = 12 mice) where 2 wounds were created per animal and treated daily either with PBS or rHDL at 75 µg from Day 0 to Day 10. (**B**) Wound closure was analyzed in diabetic C57BL/6J mice, where the daily treatment either with PBS (n = 24) or rHDL at different doses including 10 µg (n = 12), 30 µg (n = 12), and 50 µg (n = 24) per wound, started 2 days after surgery up to Day 10. (**C**) Histological and immunohistochemical analysis of wounds treated with PBS or rHDL (each dot represents one wound). Representative images (scale bars = 50 µm) and corresponding quantitative analyses are shown. Masson’s trichrome stained skin tissue (upper panels) was used to determine collagen deposition; αSMA as marker for myofibroblasts; anti-mouse Ly6G/Ly6C to determine neutrophil infiltration, and anti CD68 as macrophage marker. Data are presented as mean ± SD. * *p* < 0.05, ** *p* < 0.01, *** *p* < 0.001, **** *p* < 0.0001 depicted are based on comparisons against PBS group. αSMA, alpha-smooth muscle actin; PBS, phosphate-buffered saline; rHDL, reconstituted high-density lipoprotein; SD, standard deviation.

We observed that topical application of 75 µg of rHDL (apoA-I to PC molar ratio 1:66) at the time of wounding significantly improved wound healing in diabetic mice from Day 4 to Day 10 by ~50% relative to vehicle control (Figure 1A and Figure S1A). When developing therapeutics to treat existing pathologies, it is important to understand the potential of new active ingredients in conditions where the pathological conditions are already present. In the following set of experiments, we evaluated the post-wounding therapeutic efficacy of rHDL in diabetic animals. When the treatment was applied 2 days post-injury, rHDL treatment significantly accelerated wound closure in a dose-dependent manner across 10, 30, and 50 µg rHDL, and started as early as 4 days after wounding (Figure 1B and Figure S1B).

The accelerated wound closure observed with rHDL treatment was associated with increased collagen production and an accompanying increase in myofibroblasts, as confirmed in the wound sections collected 10 days after wound surgery by increased positive Masson’s trichrome and αSMA staining, respectively. rHDL treatment also resulted in decreased neutrophil but increased macrophage infiltration in the wounds (Figure 1C).

### 3.2. Wound Closure in Diabetic Mice

#### Characterization of rHDL/20%Pluronic F-127 Gel Formulation

**Figure 2:**
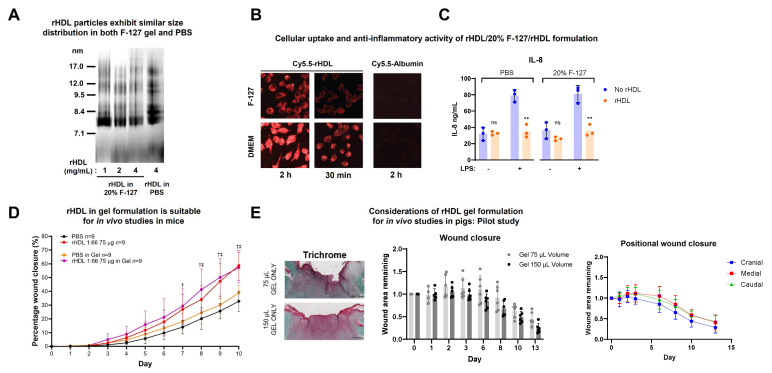
Characterization of rHDL encapsulated in 20% Pluronic F-127 gel. (**A**) rHDL formulations containing 1, 2, or 4 mg protein/mL in 20% Pluronic F-127 or PBS were analyzed by non-denaturing polyacrylamide gradient gel electrophoresis, followed by GelCode Blue staining. (**B**) Cy5.5- rHDL (1 mg protein/mL final concentration) in DMEM (lower panel) or in DMEM supplemented with 20% Pluronic F-127 gel (upper panel) was incubated with RAW 264.7 cells for 30 min or 2 h at 37 °C. Cy5.5-Albumin was used as a control. Fluorescence microscopy was used to visualize cellular uptake of Cy5.5-rHDL. (**C**) rHDL formulations containing 1 mg protein/mL in 20% Pluronic F-127 or PBS were incubated with PBMCs with or without LPS stimulation (final concentration 100 ng/mL). PBS liquid formulation or PBS in gel (20% F-127) alone were used as corresponding controls for rHDL treatment. IL-8 levels were measured in cell-free supernatants 6 h after LPS stimulation. Mean values ± SD were derived from triplicate cell cultures from three healthy donors. ** *p* < 0.01, LPS-stimulated cells treated with rHDL in PBS or 20% Pluronic F-127 vs. the corresponding PBS or 20% Pluronic F-127 control. (**D**) Wound closure was analyzed in diabetic C57BL/6J (n = 9 mice/group) treated with either rHDL or PBS formulated in liquid solution or 20% Pluronic F-127 gel for up to 10 days. † Denotes significance between rHDL (1:66) 75 µg in PBS and PBS alone; ‡ denotes significance between 75 µg rHDL in 20% Pluronic F-127 gel (1:66; rHDL in gel) and 20% Pluronic F-127 gel alone (PBS in gel). (**E**) Comparison of two volumes of 20% Pluronic F-127 gel applied to wounds in healthy pigs in terms of wound closure and macroscopic histology. In addition, wound closure was compared across different wound positions along the longitudinal axis. Data are presented as mean ± SD. DMEM, Dulbecco’s Modified Eagle Medium; IL-8, interleukin-8; LPS, lipopolysaccharide; PBS, phosphate-buffered saline; rHDL, reconstituted high-density lipoprotein; SD, standard deviation.

After confirming the potential of a liquid formulation of rHDL in PBS to accelerate wound closure in diabetic mice under therapeutic conditions (i.e., starting the treatment two days after wounding), and considering the need for a gel formulation for application in patients, we prepared a formulation of rHDL in a thermo-reversible 20% Pluronic F-127 hydrogel. Pluronic F-127 hydrogel forms a thermosensitive hydrogel that can be used to encapsulate therapeutic agents, enabling site-specific delivery to wounds and improving local concentration while reducing systemic side effects [49]. To evaluate the new gel formulation while minimizing additional experimental variables introduced by delayed treatment initiation, treatment was started immediately after wounding in this proof-of-concept formulation study. This approach allowed us to directly assess the performance of the Pluronic F-127 formulation under standardized wound healing conditions before advancing to the more translational porcine model. To characterize rHDL/20% Pluronic F-127 gel formulation, the following parameters were assessed: particle size distribution, ability to penetrate the cells, and anti-inflammatory function. Figure 2A shows that rHDL encapsulated in 20% Pluronic F-127 gel at different protein concentrations (1, 2, or 4 mg/mL) exhibits a similar particle size distribution as assessed by non-denaturing polyacrylamide gradient gel electrophoresis. The uptake of fluorescently labeled Cy5,5-rHDL from Pluronic F-127 gel by murine macrophage RAW264.7 cells was slower compared to that of Cy5.5-rHDL solubilized in PBS at each timepoint (30 min and 2 h, Figure 2B). This observed reduction in uptake may be attributed to the restricted mobility of particles in the gel matrix, which limits their availability at the cell surface for internalization. The anti-inflammatory functions of the two rHDL formulations, encapsulated in Pluronic F-127 gel and solubilized in PBS, were similar. Both rHDL formulations completely inhibited IL-8 release from LPS-stimulated PBMCs (Figure 2C).

The efficacy of Pluronic F-127/rHDL was evaluated in the diabetic mouse model and compared to the liquid formulation. rHDL in both gel and liquid formulations accelerated wound closure in diabetic mice. Notably, the gel formulations (i.e., “PBS in gel” and “rHDL in gel”) showed a modest advantage over the liquid formulations (Figure 2D and Figure S1C).

### 3.3. Testing the Pluronic F-127/rHDL Formulation in Healthy Pigs

In a pilot study on healthy pigs with excisional wounds, we determined the appropriate volumes of the gel formulation and tested lower concentrations of rHDL, to define a lower range of rHDL, with no apparent effect on wound healing parameters (Figure 2E, Figures S1D and S2A). We used the data from the pilot study to compare the wound closure between different anatomical regions (Figure 2E, right panel) as well as with data from other studies to determine the sources of variance in primary wound healing outcomes (i.e., wound closure). The main source of variance was the animal itself rather than the region of the wound.

### 3.4. Wound Closure in Diabetic Pigs

#### Topical Administration of rHDL Gel Formulation Failed to Improve Wound Closure in Diabetic Pigs

Following the demonstration of rHDL efficacy in mouse models of wound healing and the development of rHDL gel formulation suitable for in vivo studies in larger animal models, we advanced preclinical translational studies to evaluate the efficacy of Pluronic F-127/rHDL in promoting wound healing in diabetic Yucatan miniature pigs. After more than 2 months of confirmed alloxan-induced diabetes (Figure S1E), eight pigs underwent surgery to create eight full-thickness excisional wounds per animal. To achieve a balanced distribution of treatments per animal and wound site, we allocated between 10 and 11 wounds per treatment, ensuring that each animal had at least one wound for each treatment (Figure S2B). This approach was adopted based on our pilot studies, which identified the individual animal as the main source of variation.

**Figure 3:**
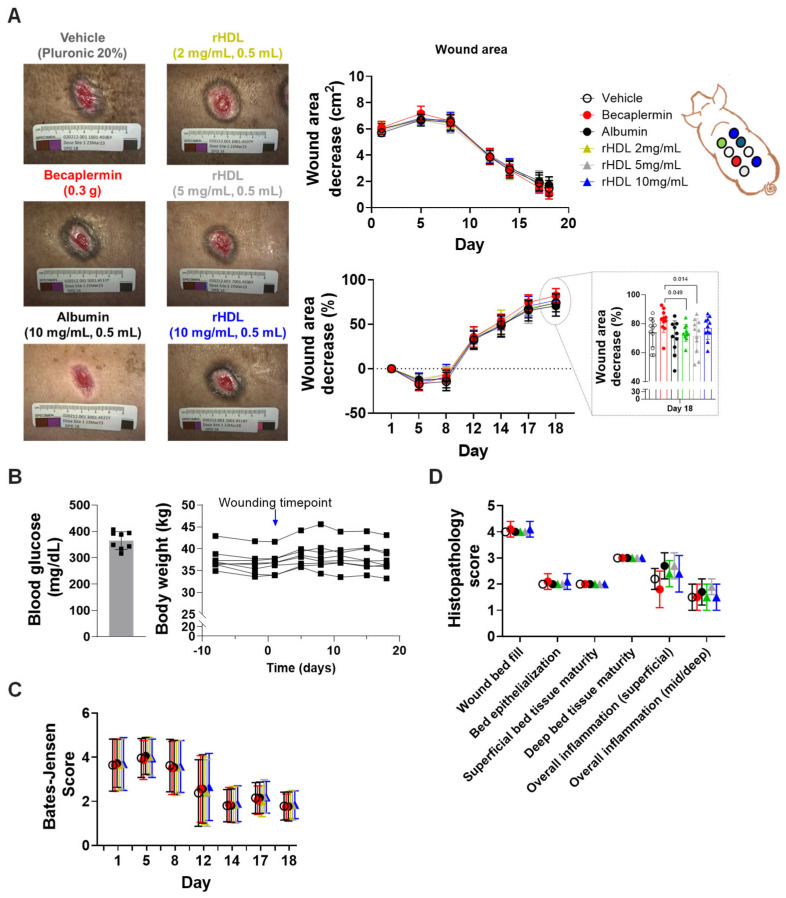
Topical daily administration of the rHDL gel formulation failed to improve wound closure in diabetic pigs. (**A**) Wound closure was evaluated by reduction in wound area over time, with representative images shown for Day 18. Diabetes was confirmed by persistently elevated glucose levels, while body weight remained stable throughout the study period. (**B**) The inset shows the decrease in wound area on Day 18, the final day of observation. The amount of applied becaplermin is described in the materials and methods and was calculated based on the initial wound size (4.9 cm^2^). *p*-values were derived from comparisons of marginal means generated using the generalized least squares (GLS) model with single-step correction for multiple comparisons [46]. (**C**) Macroscopic wound healing assessed using the Bates-Jensen dermal scoring. (**D**) Histopathological scoring of wound tissue sections. Data are presented as mean ± SD. rHDL, reconstituted high-density lipoprotein; SD, standard deviation.

We tested three concentrations of rHDL in comparison to a vehicle control of 20% Pluronic F-127 in PBS, and the reference treatment with becaplermin (PDGF-BB), which is available as a topical gel (Figure 3). Wound closure, assessed by histopathology and molecular analysis, was similar between all the tested treatments at all observed time points. Importantly, diabetes was evident in all animals with elevated blood glucose levels observed on Day 18; there were no changes in body weight during the study (Figure 3B). Compared to vehicle control, rHDL did not demonstrate a significant enhancement in wound closure at any observation time point up to Day 18. The highest rHDL concentration tested (10 mg/mL) resulted in an adjusted MD of 3.44% (95% CI: −5.46, 12.33; *p* = 0.691) in wound area reduction compared to vehicle control. The effect of lower concentrations was negligible, and the narrow confidence intervals indicate that a treatment effect greater than a 12% increase in wound closure can be ruled out. In contrast, on Day 18, the benchmark comparator, becaplermin (0.3 g), significantly accelerated wound closure when compared to 2 mg/mL (MD of −9.13%; 95% CI: −18.25, −0.02; *p* = 0.049) and 5 mg/mL rHDL (MD of −10.65%; 95% CI −19.55, −1.75; *p* = 0.014). Albumin, used as an additional control, had no apparent effect on wound closure rate (Figure 3A and Table S2).

Macroscopic assessment of wound healing using the Bates-Jensen score was also comparable between treatments on Day 18 (Figure 3C). Microscopic histological analysis, which assessed wound healing characteristics including wound bed filling, bed epithelialization, superficial and deep bed tissue maturity, and overall inflammation at the superficial, mid, and deep levels, showed no significant differences between treatments on Day 18. Interestingly, there was a trend toward reduced superficial inflammation in the becaplermin-treated wounds, indicating greater epithelialization of the wounds (Figure 3D).

### 3.5. Wound Healing in Diabetic Pigs

#### Topical Administration of rHDL Does Not Improve Quantitatively Histopathological Features of Wound Healing in Diabetic Pigs

To further extend the histological characterization of the rHDL-treated wounds, we performed a second independent histopathologic analysis using the ISO 10993-6:2016 score for item performance on wound healing. Overall, the tissue defect was completely filled with granulation tissue in all wound sites. The morphology of the granulation tissue varied between wound sites, from a predominance of horizontal collagen fibers with a fascicular pattern and reactive fibroblasts to a mature collagenous matrix with horizontal fascicles of collagen fibers and fibrocytes with perpendicularly arranged vessels. Re-epithelialization levels of the tissue defect varied between wound sites within the same group and between groups. Partial coverage was observed in the 2 mg/mL and 5 mg/mL rHDL groups and the albumin control; more extensive coverage of the defect was present in the becaplermin and vehicle groups. Overall, becaplermin showed the most favorable wound healing parameters with mature granulation tissue and extensive re-epithelialization of the tissue defect and the lowest host reaction score across the different skin layers. The lowest wound healing scores were seen in the 2 mg/mL and 5 mg/mL rHDL groups, with incomplete re-epithelialization and higher host reaction scores due to higher numbers of inflammatory cells (Table 1, Tables S3 and S4).

**Table 1:** Histological cutaneous parameters for the assessment of wound healing performance: Summary of wound healing parameter scores.

Host Reaction at Wound Site	Vehicle (PBS)	Becaplermin	Albumin 10 mg/mL	rHDL 10 mg/mL	rHDL 5 mg/mL	rHDL 2 mg/mL
**Number of samples**	11	11	10	11	10 *	10
**Re-epithelialization**	3.0 ± 0.6	3.2 ± 0.8	2.5 ± 0.7	2.9 ± 0.7	2.5 ± 0.5	2.2 ± 0.6
**Granulation tissue (defect tissue replacement)**	4.0 ± 0.0	4.0 ± 0.0	4.0 ± 0.0	4.0 ± 0.0	4.0 ± 0.0	4.0 ± 0.0
**Collagen maturation**	4.0 ± 0.0	3.8 ± 0.4	4.0 ± 0.0	3.8 ± 0.4	3.9 ± 0.3	4.0 ± 0.0
**Serocellular crust**	0.3 ± 0.5	0.5 ± 0.5	0.5 ± 0.5	0.4 ± 0.5	0.8 ± 0.07	0.4 ± 0.5

Data are presented as mean ± SD. All *p* > 0.05 based on comparisons of active treatments with PBS vehicle group (see Table S6). Refer to Table S5 for the definition of the scores. * Sample 7 from animal number 4 was excluded due to processing artifacts. PBS, phosphate-buffered saline; rHDL, reconstituted high-density lipoprotein.

**Figure 4:**
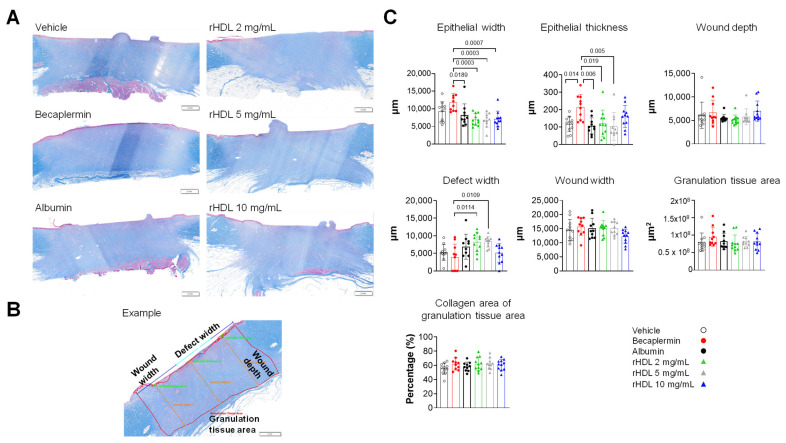
Topical daily administration of rHDL does not improve histopathological parameters of wound healing in diabetic pigs. (**A**) Representative images of wounds from each treatment group stained with Masson’s trichrome (scale bars = 2 mm). (**B**) Overview showing wound width, defect width, epithelial thickness, wound depth, and granulation tissue area. (**C**) Quantitative analysis of wound healing parameters was performed using Olympus imaging and image analysis software, cellSens v3.1. The presented *p*-values are based on one-way ANOVA and *post hoc* Tukey’s multiple comparisons test. Data are presented as mean ± SD. rHDL, reconstituted high-density lipoprotein; SD, standard deviation.

Histomorphometry analysis of tissue regeneration/integrity characteristics showed that the becaplermin group also had significantly greater epithelial width (length of regenerated epithelium) and greater epithelial thickness (thickness/height of regenerated epithelium) compared to most other treatments. Additionally, becaplermin resulted in significantly less width defect compared to rHDL 2 and 5 mg/mL treatments (Figure 4). Other analyzed wound tissue parameters, such as wound depth, wound width, granulation tissue area, and collagen area of the granulation tissue area, were not significantly different between treatments (Figure 4). The applied rHDL doses in diabetic pigs (10.2, 5.1, and 2.04 µg/mm^2^) overlapped with or exceeded the highest doses tested in mice (2.65 and 1.77 µg/mm^2^), indicating that the lack of efficacy in pigs was not due to insufficient topical exposure. In summary, rHDL showed no improvement of wound healing after topical application in the diabetic pig model.

## 4. Discussion

DFUs are a common serious complication of diabetes, leading to significant morbidity and mortality, and despite the availability of various therapeutic approaches, many ulcers become chronic and fail to heal [4,5,6,7,8,10,11]. Impaired wound healing under diabetic conditions is a result of complex and diverse pathological mechanisms, with persistent unresolved inflammation being a central feature of the disease [6,12,13,14,15,16]. The antioxidant and anti-inflammatory properties of HDL and its role in tissue repair and wound healing in mouse models of metabolic diseases make HDL a promising candidate for the treatment of diabetic wounds [29,30,31,35,36,37,38,39]. The efficacy of rHDL in improving wound healing has been demonstrated in mouse models, including diabetic and high cholesterol models, when HDL was applied concurrently with wound creation [35,36,37,38,39]. However, its efficacy on wound healing when applied to existing wounds has not been previously evaluated, prompting our evaluation of this hypothesis.

This study successfully demonstrated the efficacy of rHDL to improve wound healing when applied both early and 2 days post-wounding in a diabetic mouse model. rHDL enhanced wound healing by promoting collagen production, and by modulating inflammation. As a next step, applicable to the development of topical drugs for wound healing, we tested the efficacy of rHDL in a diabetic pig model [21,50,51,52,53]. An advantage of porcine models of wound healing, in addition to the similarity in physiology and anatomy to human skin, is the ability to have multiple wounds in one animal that can be treated differently, allowing the use of fewer animals. However, topical application of rHDL did not improve wound closure or any macroscopic and microscopic parameters of wound healing. These findings do not support the potential use of a topical rHDL formulation in the treatment of DFUs.

The development of new treatments for wound healing is impeded by several challenges, including the lack of understanding of the healing process’s complexity, interspecies differences, and limitations associated with preclinical models. Preclinical experiments are predominantly performed in rodents, whose wound healing mechanisms differ substantially from those of pigs and humans [21,51]. Therefore, the differences between the mouse and porcine data were anticipated. Humans and pigs demonstrate similar wound healing processes, primarily occurring through re-epithelialization and granulation tissue formation, whereas in mice, wound healing occurs primarily by wound contraction, which can be reduced using silicone splints. Due to the closer similarities between human and porcine skin compared to mice, a pig model was selected as the relevant species for further validation and evaluation of rHDL [54,55,56,57,58]. Pigs exhibit a lipid milieu similar to humans, with higher LDL-cholesterol and complex HDL profiles compared to mice [59,60,61]. These differences could potentially influence the effect of rHDL; however, we took this into account by using higher concentrations of rHDL in pigs than in mouse studies. Further mechanistic exploration would require dedicated comparative research beyond this work. Therefore, we believe that the study in diabetic pigs was carefully designed and conducted to allow appropriate decision-making; however, our findings failed to support a topical rHDL approach for treatment of diabetic wounds.

Additional confidence in the porcine study is based on our observation that becaplermin promoted better wound healing (e.g., enhanced re-epithelialization) compared to all other groups. This was observed despite becaplermin being administered once daily in the present study, whereas clinical administration is typically performed twice daily. The once-daily dosing schedule was selected due to technical and logistical constraints associated with the experimental protocol. Furthermore, the main effects of becaplermin reported in preclinical models [62] relate to increased granulation tissue formation, which is observed at early time points (Day 7 in non-diabetic animals). In this study, we observed only a non-significant increase, likely due to the later observation time point (Day 18), at which re-epithelialization was already nearly complete.

Gel formulation was selected not only to allow easy application and targeted delivery, but also for its intrinsic cutaneous properties by maintaining a moist environment. However, the modest improvements in wound closure observed with the gel formulation were likely due to the Pluronic F-127 gel itself, an observation that has been previously reported [63]. In addition, a probable limitation of our study is that it was not possible to achieve higher concentrations of rHDL (beyond 10 mg/mL) in gel formulation. Pluronic gels are known to have a beneficial effect on wound healing [63,64] that may be further enhanced by the addition of a potentially active therapeutic, such as rHDL. However, the concentrations of rHDL used in this study were higher than those administered in the mouse model or in other studies (including those dosing every 2 days) [35,37,38], making it a less plausible explanation for the lack of observed effect. Furthermore, we do not exclude that there could be a beneficial effect of systemic application of rHDL on wound healing by reducing global and local inflammation, as proposed by regulating endothelial cell activation in a porcine model of acute cutaneous inflammation, where rHDL was applied systemically and reduced inflammatory markers [65]. A constraint of the present study is that direct assessment of rHDL penetration, retention, stability, cholesterol efflux activity, or local pharmacodynamic activity within porcine wound tissue was not performed. However, in vitro characterization confirmed preservation of rHDL structural integrity and biological activity in both PBS and Pluronic F-127 gel formulations prior to topical administration; differences in tissue permeability, wound microenvironment, wound exudate composition, or local retention between murine and porcine skin may have influenced effective exposure at the wound site. Therefore, reduced tissue penetration, altered local bioavailability, or impaired biological activity of rHDL in the porcine model cannot be excluded as a contributing factor to the lack of observed efficacy. Notably, the porcine studies were designed to maximize local wound exposure through immediate treatment initiation, daily topical administration, and higher rHDL doses compared with the murine studies; however, no therapeutic benefit was observed under the experimental conditions tested.

Another limitation of our study is that induction of diabetes with streptozotocin or alloxan produces a phenotype similar to type 1 diabetes [66], while the majority of diabetic patients have type 2 diabetes associated with obesity and additional metabolic comorbidities. Obesity contributes to impaired wound healing through several mechanisms, including excess adipose tissue accumulation, chronic low-grade inflammation, altered adipokine signaling, vascular dysfunction, tissue hypoxia, and disruption of skin homeostasis [67,68,69]. These obesity-associated alterations are not fully reproduced in the experimental models used in the present study. However, molecular mechanisms associated with impaired wound healing (e.g., chronic inflammation, increased reactive oxygen species levels) are common to both types of diabetes; therefore, findings from these models might still be relevant. In this regard, several approaches have been developed to create animal models that closely resemble the patient situation; however, it is challenging to replicate the significant heterogeneity of ulcers observed in patients with diabetes [32]. Furthermore, although excisional wound models are widely used and well established in diabetic wound healing research, they do not fully reproduce all pathological features of human diabetic foot ulcers, including chronic pressure-induced tissue damage and ischemia. Pressure-induced wound models may, therefore, provide additional clinical relevance for future studies evaluating rHDL or related therapeutic approaches. Nevertheless, excisional diabetic wound models have historically served as important translational platforms for preclinical evaluation of wound therapeutics prior to clinical development. The diabetic-induced miniature swine is a well-accepted, non-rodent model for dermal wound healing studies due to the anatomical similarities between swine and humans, including skin thickness, subcutaneous (s.c) tissues, and underlying muscular structures. For this reason, the Yucatan miniature pig is considered an appropriate species for evaluating topical drugs and the wound healing properties of therapeutic candidates [52,70]. Additionally, dermal wounds produced in these animals mimic a full-thickness skin lesion consistent with worst-case clinical scenarios [52,70]. Although we used a single sex in mice and pigs for the efficacy testing, we acknowledge that sex and sex hormones may influence wound repair, for example, as evidenced by sexual dimorphism in rHDL-induced angiogenesis in mice [38]. In case of a positive efficacy outcome, additional studies in the other sex would have been warranted; however, given the lack of efficacy under the conditions tested, further animal use was not scientifically justified. As with any model, there are some well-known limitations; however, if these are well considered, the models play a critical role in establishing pharmacological responses and assessing potential toxicities of wound treatment products, especially when other alternatives, such as in vitro models, do not offer the complex physio- and patho-physiological environment and synergies of the whole organism [50,51,52,58]. To further characterize the diabetic pig model used, and to monitor animal health status, hematology, serum chemistry, and coagulation profiles were analyzed 3 days before wounding and again on day 18. These data (Table S1) remained within the expected reference ranges for miniature swine [42] and did not exhibit abnormalities typically associated with diabetic ketoacidosis (DKA) or hyperosmolar hyperglycemic state (HHS). Specifically, hematocrit values remained within normal ranges, arguing against hemoconcentration associated with significant dehydration. Serum electrolytes (Na^+^, K^+^, and Cl^−^) also remained within normal ranges for miniature swine, without patterns suggestive of metabolic acidosis, dehydration, or electrolyte imbalance typically observed in DKA or HHS. Furthermore, the animals showed no hypernatremia, no mental status alterations, and no laboratory evidence suggestive of hyperosmolarity, which are key diagnostic features of HHS. Coagulation parameters were also within normal ranges, and necropsy revealed no macroscopic abnormalities. Taken together, the clinical observations, stability of body weight and hydration status, and the hematology, serum chemistry, and coagulation data provide no evidence that the diabetic porcine model was compromised by manifestations associated with DKA and/or HHS.

At the histological level, rHDL showed no improvement over the controls, including the reference treatment, becaplermin, which demonstrated superior wound healing parameters and the lowest host reaction score [51]. Future research in wound healing should focus on larger patient cohorts in randomized prospective clinical trials, complemented by registry and retrospective studies. This approach will help clinicians to identify patients who will most likely benefit from specific treatment modalities and to support further design of preclinical studies. At the same time, research should focus on making these therapies cost-effective and accessible. Additional research avenues may include personalized approaches that tailor treatments to specific patient needs based on wound severity, comorbidities, and genetic factors. Collectively, these efforts will be critical in addressing the ongoing burden of DFUs, ultimately improving patient outcomes and reducing healthcare costs [12,53].

## 5. Conclusions

We conclude that administration of rHDL enhances wound closure in a murine model of diabetic wound healing by promoting collagen production and modulating inflammation. However, despite these beneficial effects observed in mice, topical rHDL formulated in Pluronic F-127 gel failed to improve wound healing outcomes in the more translationally relevant diabetic porcine wound healing model under the experimental conditions tested. These findings highlight the challenges associated with the translation of therapeutic efficacy across preclinical wound healing models and emphasize the importance of evaluating candidate therapies in physiologically relevant systems prior to clinical development. Finally, a general challenge is the complexity of wound healing; an optimal therapy would promote repair of the various layers of damage within the wound bed while simultaneously initiating multiple critical signaling events, reducing inflammation, and promoting tissue repair, including healthy wound closure and neovascularization to provide oxygen and nutrients. To achieve this, multi-mechanistic approaches, drug repurposing, and carefully designed research programs, including refined and translationally relevant models, should be considered.

## Data Availability

The original contributions presented in this study are included in the article/Supplementary Materials. Further inquiries can be directed to the corresponding author.

## Supplementary Materials

The following supporting information can be downloaded at: https://www.mdpi.com/article/10.3390/biom16071001/s1, Supplemental Methods; Table S1: Summary of hematology, serum chemistry and coagulation parameters; Table S2: Wound area reduction on Day 18 (differences in mean response with 95% CI); Table S3: Summary of local effects/host reaction based on the average full score; Table S4: Summary of ISO 10993-6 scores across the different treatment groups; Table S5: Histological cutaneous parameters for the assessment of wound healing performance; Table S6: Summary of Hodges-Lehman effect estimates and *p*-values corresponding to Table 1; Figure S1: Absolute wound size, body weight and blood glucose; Figure S2: In vivo characterization of rHDL gel formulation suitable for studies in pigs. Major Resources Table. ARRIVE study plans.

## Abbreviations

The following abbreviations are used in this manuscript:

αSMA	alpha-smooth muscle actin
ANOVA	Analysis of variance
apoA-I	Apolipoprotein A-I
apoE	Apolipoprotein E
CI	Confidence intervals
DFUs	Diabetic foot ulcers
DMEM	Dulbecco’s Modified Eagle Medium
FCS	Fetal calf serum
GLS	Generalized least squares
H&E	Hematoxylin and eosin
HDL	High-density lipoprotein
IACUC	Institutional Animal Care and Use Committee
IL-8	Interleukin-8
i.m.	Intramuscular
i.p.	Intraperitoneal
i.v.	Intravenous
INHAND	International Harmonization of Nomenclature and Diagnostic Criteria
ISO	International Organization for Standardization
LPS	Lipopolysaccharide
MD	Mean differences
PBMC(s)	Peripheral blood mononuclear cell(s)
PC	Phosphatidylcholine
PBS	Phosphate-buffered saline
PDGF	Platelet-derived growth factor
POD	Post-operation day
PVC	Polyvinyl chloride
rHDL	Reconstituted high-density lipoprotein
RITA	Registry of Industrial Toxicology Animal-data
s.c	subcutaneous
SD	Standard deviation
SE	Standard error
STP	Society of Toxicologic Pathology
TBST	Tris-Buffered Saline/0.1% Tween 20
*w/v*	Weight/volume
