# Supplementary material for: Evaluation of Topical Reconstituted HDL as a Treatment for Diabetic Wounds in Murine and Porcine Models
Source: Biomolecules. 2026 Jul 9;16(7):1001. doi: 10.3390/biom16071001 (PMC13407075; doi:10.3390/biom16071001)
Supplement: Supplementary file 1 [file biomolecules-16-01001-s001.zip › ARRIVE study plans.pdf]

ARRIVE study plan

Please fill in all sections of the ARRIVE study plan.

Study details

Study title:	Wound healing in diabetic mice	Grant code:	N/A
Start date:	23/07/2020	End date:	07/07/2022
Project licence or permit number:	#2011	Project lead:	Helen Cao
Protocol numbers:	BioSOP001- Euthanasia of mice via CO_2 asphyxiation;BioSOP002 – Anesthesia of mice with isoflurane inhalation; BioSOP006 – Anesthesia of mice with ketamine/xylazine. Full-thickness excisional wound surgery.	Expected severity:	Major but brief duration.
Primary responsible:	Helen Cao	Contact details:	Helen.Cao@csl.com.au
Secondary contact:	N/A	Contact details:	N/A

Experimental animals

Species	Strain/Genotype	Sex	Age	Weight	Source	Number
Mouse	C57BL/6J	Male	8	20-23g	Animal Resources Centre, Australia	9-12 per treatment group
					Total number	132

Experimental procedures

What is done and how is it done, when and how often.

Procedures:	Diabetes was induced via a single intraperitoneal (i.p.) administration of streptozotocin (STZ) at 165 mg/kg two weeks prior to the surgery. Blood glucose levels were measured one week after STZ injection by collecting small blood samples from the tail vein using the Accu-CHEK Performa Blood Glucometer. If the glucose levels were lower than 10-12 mmol/L, a boost injection of STZ at 60 mg/kg was given. The glucose levels were measured again on Day 0 and the mice with the glucose levels of 15.0 mmol/L or above were considered diabetic and selected for wounding surgery (almost 100% mice were diabetic from the diabetic induction protocol). Wounding surgery was performed as detailed below. Following the surgery, the mice were housed in individual cages. Wound sizes were measured daily along the X, Y and Z axis using calipers after mice were anesthetized by isoflurane inhalation. 20 uL of rHDL or PBS was topically applied to the wounds daily. At the end of the study, the mice were humanely killed via CO2 asphyxiation, and wound tissues were excised and processed for histological and immunohistochemical analysis.
Surgical procedures:	For wounding surgery, mice were anesthetized with a single injection of ketamine (100 mg/kg)/xylazine (20 mg/kg). Two 6-mm circular full-thickness excisional wounds were surgically created on either side of the dorsal skin after depilation. A donut-shaped 12-mm silicon splint was then placed around the wounds and adhered to the skin with superglue and interrupted 6-0 nylon sutures. The wounds were covered with a transparent occlusive dressing. Following the surgery, the mice were housed in individual cages. Wound sizes were measured daily along the X, Y and Z axis using calipers after mice were anesthetized using isoflurane inhalation. rHDL or PBS was topically applied to the wounds daily. Analgesia was provided by a subcutaneous injection of buprenorphine at 0.1 mg/kg before the surgery and subcutaneous injections of carprofen at 4.4 mg/kg once daily for post-operative care. After surgery, Diet Recovery gel was provided on the cage floor to stimulate food consumption.
Anaesthesia:	Before wounding surgery, mice were anesthetized with a single intraperitoneal injection of ketamine (100 mg/kg)/xylazine (20 mg/kg). For daily wound size measurement and administration of rHDL or PBS, mice were anesthetized with isoflurane inhalation.
Analgesia:	Analgesia was provided by a subcutaneous injection of buprenorphine at 0.1 mg/kg before the surgery and subcutaneous injections of carprofen at 4.4 mg/kg once daily for post-operative care.
Locations:	Animal Procedure Room 602, Bio21 Institute Animal Facility, University of Melbourne, Australia
Acclimatisation period:	C57BL/6J mice were purchased from Animal Resources Centre, Australia and acclimatized to their environment for at least 1 week before the procedures.

Animal care and monitoring

Adverse events:	N/A		
Humane endpoints:	Euthanasia was immediately conducted in the animals if any of the following events occurred: body weight loss reached 20% or greater, wound infection (not noted), loss of suturing integrity or presence of significant distress (not noted) with the symptoms of hunched posture, ruffled or difficult in breath. The exclusion of the animals from the studies due to the above reasons was about 15-20%.		
Welfare monitoring:	Mice were closely monitored after each procedure until full recovered. After wounding procedure, mice were monitored until fully recovered from anaesthesia.Before wound induction, mice were monitored 3 times per week (e.g. Monday, Wednesday and Friday). However, the monitoring frequency were increased to daily if a significant weight loss is shown e.g. reaching to 15% loss. After wounding surgery, mice were monitored daily. The condition of wounds was closely monitored e.g. suturing integrity and infection. If loss of suturing integrity (i.e. the silicone splint was not secured on place to prevent wound contraction, or infection occurs (both are rare i.e. no infection was noted), mice were euthanised immediately by CO2 asphyxiation. Mice would lose some body weight after diabetes induction. Any animals with 20% or greater weight loss were euthanized immediately by CO2 asphyxiation. Mice were also monitored for signs of lethargy, ruffled fur, hunched gesture. Any animals with any of these signs were monitored every 1-2 hours and euthanasia was conducted if the signs persisted for 4 hours. Any animals with signs of difficulty in breathing were euthanized immediately.	Attached clinical assessment form:	No
Changes in housing and husbandry:	After wounding surgery, mice were singly housed in order to eliminate social grooming/cleaning behavior resulting in chewing off the fine suture material and silicone splint.		
Restrictions in veterinary care:	N/A		

Risks

Emergency procedures:	N/A
Potential risk to personnel:	N/A

Personnel involved in the experiment		
Sharelle Sturgeon	Wound surgery, drug administration, wound size measurement, animal monitoring and euthanasia, tissue harvest after euthanasia.	Trained and competent☒
Yun Dai	Animal monitoring and euthanasia, tissue harvest after euthanasia. IHC and analysis.	Trained and competent☒

Study design and sample size

Experimental groups:	In the studies in Figure 1, the difference in wound closure between rHDL and PBS treatment groups was compared; In the studies in Figure 2, the difference in would closure between rHDL in liquid and PBS or between rHDL in gel and gel solution was compared.		
Experimental unit:	Mice	Sample size per group:	9-12
Justification for sample size:	A sample size of n=9-12 mice are included from the outset. These group sizes are informed by historical data and practical experience rather than formal sample size calculations.		
EDA read only diagram:	N/A	Access code:	N/A

Inclusion and exclusion criteria

Inclusion criteria:	Mice with glucose levels at 15 mmol/L or more (i.e. diabetic) without significant body weight loss or signs of distress were selected for wounding surgery.
Exclusion criteria:	The mice with the glucose levels lower than 15 mmol/L, body weight loss reaching 20% or greater, loss of suturing integrity, wound infection or signs of significant distress were removed from the studies.
Expected attrition:	Approximately 15-20% of the mice in the diabetic wound healing group are expected to be excluded due to STZ-induced weight loss and loss of suturing integrity with the potential need for euthanasia.

Randomisation and blinding/masking

Method of allocation to group:	Randomization was stratified by weight, cage and glucose levels such that mice were allocated into different treatment groups.
Strategy to minimise confounders:	Same sex and age of mice were used (with similar body weight); mice were randomized and allocated into treatment groups; same housing was implemented; standardized humane handling techniques were conducted to reduce stress and anxiety to animals; procedures were staggered e.g. anesthesia, wound size measurement, rHDL/control administration in the way to ensure that the duration of the treatment is the same across all animals.

Blinding strategy:	Randomization was implemented to allocate mice into treatment groups; due to the complexity of the wounding surgery and following procedures, it is impractical to implement topical application of treatments into wounds and assessment of wound closure; all histological and immunohistochemistry analysis was conducted in a blinded manner.

Outcome measures and statistical methods

Outcome measures:	Primary Outcome:<br><br>Wound healing progression in diabetic mice, assessed by daily measurement of wound size along the X, Y, and Z axes using calipers over a 10-day period.<br><br>Secondary Outcomes:<br><br>Histological and immunohistochemical analysis of wound tissues at study endpoint to evaluate tissue regeneration and inflammatory markers<br><br>Blood glucose levels measured using the Accu-CHEK Performa Blood Glucometer to confirm diabetic status (≥15.0 mmol/L).
Primary outcome measure:	Wound healing progression in diabetic mice, assessed by daily measurement of wound size along the X, Y, and Z axes using calipers over a 10-day period.

Analysis plans:	For the therapeutic treatment study, i.e., rHDL and PBS administration commenced two days after wound surgery, data were expressed as mean ± standard deviation (SD) of the percentage wound closure. Differences between PBS and treatment groups were analyzed using a linear mixed-effects model. The model included mouse as a random effect to account for correlation between multiple wounds within the same animal and repeated measurements over time. Dunnett's multiple comparison test was applied to assess differences between rHDL and PBS. For the prophylactic treatment study, rHDL and PBS administration commenced on the same day as wound surgery, data were expressed as mean ± SD of the percentage wound closure as described above. Differences between PBS and rHDL treatment were analyzed using a linear mixed-effects model with between-subject treatment effects. The model included mouse as a random effect to account for correlation between multiple wounds within the same animal and repeated measurements over time. For the histological and immunohistochemical analysis of wounds, the nonparametric Mann–Whitney U test was used to compare the percentage of αSMA-positive area, CD68-positive area, collagen-positive area, and neutrophil-positive area between the rHDL and vehicle groups. The group sizes were informed by historical data and practical experience rather than formal sample size calculations. For the murine studies, the mouse was considered the experimental unit.Statistical significance was set as $p<0.05$.

Sign off			
Primary responsible:	N/A	Date	☐ I confirm I am aware of my responsibilities as the primary responsible (e.g. conditions of the personal licence) and my training and competency record is up to date.
Project lead:	N/A	Date	☐ I confirm I am aware of my responsibilities as a project lead/licence holder and this work is in line with the project.
Internal sign off:	N/A	Date	Role, e.g. NACWO

ARRIVE study plan

Please fill in all sections of the ARRIVE study plan.

Study details

Study title:	PDS_21-01_26	Grant code:	Not applicable
Start date:	25/10/2022	End date:	23/03/2023
Project licence or permit number:	020212.001 as per Altasciences protocols	Project lead:	Scott E Boley, Joseph Agolory
Protocol numbers:	020212.001 as per Altasciences protocols	Expected severity:	Moderate
Primary responsible:	Scott E Boley	Contact details:	sboley@altasciences.com
Secondary contact:	Juan Camacho Londono	Contact details:	Juan.camacholondono@cslbehring.com

Experimental animals

Species	Strain/Genotype	Sex	Age	Weight	Source	Number
Pig	Yucatan miniature	Male	11.7-12	33.9-41.6 kg	SinclairResearch Center, Missouri, USA	8
					Total number	8

Experimental procedures

What is done and how is it done, when and how often.

Procedures:	Prior to enrollment, animals were castrated, diabetes was induced by alloxan administration to achieve fasting blood glucose levels of 150 mg/dL or above, and catheters were implanted. Hyperglycemia was regulated by insulin (Humalog and Lantus, or equivalent). Insulin and/or feed amounts were adjusted as needed to maintain acceptable blood glucose levels. Wounding surgery was performed in animals after two months of confirmed alloxan-induced diabetes. Animals were anesthetized to induce wounds. Each animal had eight wound sites (one row of four sites per side) with a diameter of 2.5 cm (~5 cm2), and appropriate depth (full thickness) spaced at least 3 cm apart. Clinical blood parameters were monitored during the acclimation (prior to randomization) and prior to termination (end: 17 days after wound creation). Sixty-four (64) wound sites from the dorsal skin of 8 diabetic Yucatan miniature castrated male swine were created, dosed (daily topical administration) and assessed for the efficacy and local tissue reaction after 17 days of treatment with 3 Test Articles, 2 Control Articles and Vehicle alone . The endpoints included body weight, clinical observations, hematology, coagulation, serum chemistry, wound observations (total modified Bates-Jensen scores), and wound planimetric analysis [Wound Area Decreasing (WAD)]. Gross observations were recorded, and tissues were collected for microscopic evaluation on Dosing Phase Day 18.
Surgical procedures:	-Full Thickness Wound Creation. A surgical scrub of each animal's skin was performed prior to wound creation. The full thickness wounds were created using aseptic surgical technique. Wound sites were placed along a paraspinal column/each side with efforts made to keep the column between the crest of the shoulders and the ilium. Each animal had eight (8) (one row of four sites/side, ~2.5 cm diameter wound sites, spaced at least 3 cm apart to the appropriate depth (full thickness). All excised tissue from wound sites were discarded. Following creation of wounds, the wound bed was cleaned with sterile saline and/or gauze to remove any foreign matter/loose tissue debris, if necessary. -Hemostasis. Frequency: As needed during wound creation. Direct pressure and/or a sterile epinephrine-saline mixture was utilized to obtain hemostasis. Cautery was discouraged but may be used in the event of excessive bleeding. Wounds that required the use of hemostatic agents or cautery were documented in the study data. Test materials were applied at least 5 minutes of reaching hemostasis.-Dressing Application. Frequency: Once following each dose application, or as needed when soiled. Each wound site was covered with a barrier dressing of nonadherent sterile gauze and transparent film. Once all sites were dosed and barrier dressing applied, the entire wound area was covered with a layer of foam pad and tear-resistant mesh (or stockinette) to prevent dislodgment of dressing materials.-Dressing Removals. Frequency: Once prior to each dose application, or as needed when soiled.If needed, the area around the wounds and/or dressing material should be lightly moistened with sterile saline to aid in dressing removal to prevent the likelihood of tissue tearing or bleeding. If any excessive exudates, dressing debris, remaining gel (if any) or tissue build up on the wound bed, efforts were made to remove using sterile materials (i.e., saline moistened sterile gauze) and avoid disturbing the surface of the wound. Once removed, all soiled dressings were discarded and the skin around the wound site was cleansed with 70% alcohol.
Anaesthesia:	Tiletamine/zolazepam (2–6 mg/kg, i.m.), xylazine (0.4–1.2mg/kg, i.m.) and atropine (0.04–0.05 mg/kg, i.m.) to produce the wounds. Animals undergoing anesthesia for surgery procedures, dose administration, and dressing changes were food fasted for at least 8 hours. Anesthesia was induced and/or maintained with isoflurane.

Analgesia:	For analgesia buprenorphine ER (extended release) (0.2–0.3 mg/kg, s.c.) and carprofen (2.2–4.4 mg/kg i.m.) were included. Cefazolin (20 mg/kg, i.v.) and Excede® (5 mg/kg, i.m.) were administered at the time of procedure for prophylaxis.
Locations:	Single-housed. Stainless steel with self-spanned polyvinyl chloride (PVC)-coated expanded metal flooring. Appropriate temperature maintained in housing room was based on animal age. >6 mos.: 61-81°F (16 – 27°C) An automatic lighting system will be set to provide a 12-hour light/dark cycle, except during designated procedures.
Acclimatisation period:	The acclimatization phase was adjusted based on the expected change of body weight after diabetes induction. The acclimation phase was extended up to Day 40, animals placed on twice daily Insulin administration, and feeding and blood glucose monitoring were performed twice daily, as normal for advanced diabetics. Body weights were monitored twice weekly. By acclimation phase Day 27, all animals had gained more weight and had improved body condition scores. Surgery was performed after 40 days of acclimation.

Animal care and monitoring

Adverse events:	There were no treatment-related effects on clinical observations, body weights and body weight changes, hematology, coagulation, and serum chemistry parameters, or macroscopic findings observed.
Humane endpoints:	Not applicable/not observed. Daily examinations were performed and several health parameters (including BCS) were considered (see above and Materials and Methods). In the event of adverse events or signs of health deterioration, a team of animal caretakers and veterinarians would determine the level, and together with the study director, decide whether endpoints had been reached.

Welfare monitoring:	Veterinary care was available throughout the course of the study and animals were examined by veterinary staff as warranted by clinical signs, including body conditioning scoring (BCS). All veterinary examinations were documented in the study data. In the events were animals required veterinary intervention, the responsible veterinarian made initial recommendations about treatment of the animal and/or alteration of study procedures.-Mortality/Morbidity check. Frequency: Twice daily: In-cage observations was made at least twice daily in the AM and PM (with at least 4 hours between observations) on non-dosing days during the in-life study period to assess general health, mortality or moribundity. If an animal was observed to have clinical signs, an unscheduled clinical observation was conducted and documented-Clinical Observations. Frequency: Starting on Dosing Phase Day 1, at least once daily prior to dose administration throughout the study. Animals were observed for any clinical signs of illness or reaction to the dose including dose site reactions. If an animal was observed to have clinical signs outside of the scheduled observations, an unscheduled clinical observation was conducted and documented. -Veterinary Physical Examination: Frequency: Animals were examined once during acclimation(prior to assignment to groups). Animals were given a physical examination by a Testing Facility veterinarian which includes, but not limited to, assessment of behavior, general body condition, condition of major body systems and skin/coat. Animals could be excluded from study selection based on findings of the acclimation physical exam (not required).	Attached clinical assessment form:	No
Changes in housing and husbandry:	Not applicable		
Restrictions in veterinary care:	Not applicable		

Risks

Emergency procedures:	Detailed protocol available for those involved in the study.
Potential risk to personnel:	Not expected carcinogenic/toxic risk of the material used. Data safety sheets were available. Pregnant women were excluded from the study (hands-on) as contact with used materials/drugs should be avoided.

Personnel involved in the experiment		
Name	Procedures they will conduct	Trained and competent☒
Name	Procedures they will conduct	Trained and competent☒

Study design and sample size

Experimental groups:	6 groups (distributed in 64 wounds, 8 wounds/animal)		
Experimental unit:	Wound	Sample size per group:	10-11
Justification for sample size:	Assuming the Normal distribution, estimates of the within-subjects and between-subjects variance components in the primary endpoint wound closure (%) were determined using a linear mixed effects (LME) regression model fitted to data from internal preliminary experiments. Using the resulting estimates of SD=15.3% (within) and SD=11.3% (between), power analyses were performed using LME regression models for different group sizes and effect sizes in terms of mean difference (MD) in wound closure between groups at the end-of-study time point. The analysis showed benchmark statistical power >80% with a group size of N=8 animals for effect sizes of MD=20% or greater. The calculations consider correction of the two-sided p-values for multiple comparisons between the k=3 active treatment dose groups with each of the control groups.		
EDA read only diagram:	n.a	Access code:	n.a.

Inclusion and exclusion criteria

Inclusion criteria:	The veterinarian determined the animal's health status before surgery. This included whether the animal had established diabetes. The veterinarian checked that the animal's health was stable and acceptable BCE. Wounds: complete tissue/wound sample allowing histopathological analysis.
Exclusion criteria:	Not applicable/required during the study. Nondiabetic or animals with reduced BCE would be excluded. Incomplete tissue/wound samples.
Expected attrition:	n.a.

Randomisation and blinding/masking

Method of allocation to group:	Animals were randomly assigned to one of the 8 treatment schemes. Treatment schemes within animals were balanced in such a way as to ensure that all treatments were applied to all possible anatomical positions of the wounds (see supplements).
Strategy to minimise confounders:	See above
Blinding strategy:	The operators and other personnel involved in data collection and sample analysis (e.g. histopathology) were unaware of the treatment applied. This information was only shared once the data were available, when it was needed for comparing the effects of the treatments.

Outcome measures and statistical methods

Outcome measures:	Wound area, wound closure (decreasing), score wound edges, score exudate type, score exudate amount, score granulation tissue, score epithelialization, Bates-Jensen score.Histopathology quantitative: Epithelial Thickness , Epithelial width , Wound width, Defect width, Granulation Tissue Area , Collagen Area of Granulation Tissue and Wound Depth.
Primary outcome measure:	Primary outcome is % wound closure by end of study based on raw outcome wound area in mm^2 which is measured longitudinally over the course of the study to track progress.
Analysis plans:	*See Materian and Methods: Studies in diabetic pigs*

Sign off

Primary responsible:	n.a	Date	☐ I confirm I am aware of my responsibilities as the primary responsible (e.g. conditions of the personal licence) and my training and competency record is up to date.
Project lead:	n.a	Date	☐ I confirm I am aware of my responsibilities as a project lead/licence holder and this work is in line with the project.
Internal sign off:	n.a	Date	Role, e.g. NACWO

ARRIVE study plan

Please fill in all sections of the ARRIVE study plan.

Study details

Study title:	Wound healing pilot study: Test of gel formulation	Grant code:	Enter
Start date:	03/05/2022	End date:	20/06/2022
Project licence or permit number:	M1700076	Project lead:	Craig Duvall
Protocol numbers:	1700076-02	Expected severity:	USDA Pain and Distress Level D
Primary responsible:	Craig Duvall	Contact details:	craig.duvall@vanderbilt.edu
Secondary contact:	Name	Contact details:	Email/phone

Experimental animals

Species	Strain/Genotype	Sex	Age	Weight	Source	Number
Pig	Yorkshire	Female	16-18	27-36	Oakhill Genetics	2
					Total number	2

Experimental procedures

What is done and how is it done, when and how often.

Procedures:	Non-Surgical Proceedures - Dressing Changes: Bandage changes occurred every 2-3 days, or more frequently if the bandage was torn, dislodged, or the wounds were damaged. Bandage change occurred until wounds have visibly resurfaced, typically 25-40 days post wounding. During dressing changes, laser doppler imaging occurred, and wounds may be photographed to document healing progress. Pigs received a pre-anesthetic dose of Tiletame-Ketamine-Xylazine and were maintained on isoflurane. The procedure typically is typically 30 minutes or less in duration. If the procedure lasts greater than 30 minutes, a pulse oximeter was used tomonitor HR, RR, and SPO2, and those parameters, as well as body temperture was recorded every 10-15 minutes. Old bandages were removed, wounds and surrounding skin gently cleansed, treatments were reapplied, a new pain patch applied when needed, and new bandages were also applied. NSP Imaging: LPDI imaging is a non-invasive technique to measure microcirculatory blood perfusion. If imaging is performed, it was at the same time as dressing changes and before euthanasia while the animal is under anesthesia. At that time the instrument's aperture was held perpendicular to the area of interest and at a recommended working distance of 10 to 25 cm above the area of interest. An on/off switchactivates the laser which performs a short scan of up to 2 minutes. The machine is attached to a cartthat is rolled into the room and subsequently removed from room after procedure. SS Excisional Wound Creation Surgery: Dorsal skin was shaved and disinfected using 70% ethanol, chlorohexidine 2% scrub, chlorohexidine 2% solution, and betadine washes. Under sterile conditions, full-thickness excisional wounds each with a total surface area of 2 cm2 were then created within two rows of 9 (18 wounds total), each positioned approximately 3 cm from the spine. Scalpel blades were used to cut wounds to a full thickness depth in the dorsal region of each pig. The epidermis, dermis, and underlying fat were removed to expose the fascia layer below. The depths of the wounds were measured at approximately 5-6 mm. Wounds were covered with layering's of Mepilex Transfer (Molnlycke Healthcare), Mextra absorbent pad (Molnlycke), OpSite adhesive (Smith&Nephew), MediChoice Tubular Net Bandage (Owens & Minor), and Vetwrap (3M) bandaging. The dressings were changed every other day. Wounds were treated on POD 1, 3, 6, 8, and 10. Wounds were cleaned with chlorohexidine 2% solution and saline, and then were allowed to dry prior to the application of treatments by positive displacement pipette. Excede®; Zoetis (5 mg/kg) was delivered intramuscularly as a systemic antibiotic on the day of surgery and 7 days post-surgery. Wounds were monitored and routinely evaluated by veterinary staff. The pigs were euthanized 13 days post-surgery via intravenous injection of euthasol (130 mg/kg), and wound samples were collected for histology. Wounds created on the dorsal region of the pig were treated topically with a maximum of 200 microliters per sq.cm area of wound with either sterile saline/water, human serum albumin (HSA),recombinant high density lipoproteins (rHDLs) or FDA approved Regranex, a recombinant plateletderived growth factor (rPDGF) to determine the efficacy of the treatment to augment wound healing and improve rate of wound closure. All biologic treatments were formulated at predetermined doses with sterile water, Pluronic 127, or saline before being applied to the wounds. The Pluronic 127 with or without any biologic treatment were applied to wounds in order to compare the resulting quality of wound healing and the rate of wound closure of these wounds to those wounds treated with Pluronic 127 which contain the biologic treatment. NSP Treatment Application: The first dose of respective treatments were applied either at the time of surgery or up to 24 hours after wound creation, after hemostasis has been achieved. Subsequently, active biologic treatments were applied every other day while the pig is under anesthesia to coincide with dressing changes and doppler imaging. All three procedures (application of biologics formulated with or without Pluronic 127,

	dressing changes, and doppler imaging) listed above will be performed under one anesthetic induction.
Surgical procedures:	Excisional Wound Creation Surgery: Dorsal skin was shaved and disinfected using 70% ethanol, chlorohexidine 2% scrub, chlorohexidine 2% solution, and betadine washes. Under sterile conditions, full-thickness excisional wounds each with a total surface area of 2 cm2 were then created within two rows of 9 (18 wounds total), each positioned approximately 3 cm from the spine. Scalpel blades were used to cut wounds to a full thickness depth in the dorsal region of each pig. The epidermis, dermis, and underlying fat were removed to expose the fascia layer below. The depths of the wounds were measured at approximately 5-6 mm. Wounds were covered with layering's of Mepilex Transfer (Molnlycke Healthcare), Mextra absorbent pad (Molnlycke), OpSite adhesive (Smith&Nephew), MediChoice Tubular Net Bandage (Owens & Minor), and Vetwrap (3M) bandaging. The dressings were changed every other day. Wounds were treated on POD 1, 3, 6, 8, and 10. Wounds were cleaned with chlorohexidine 2% solution and saline, and then were allowed to dry prior to the application of treatments by positive displacement pipette. Excede®; Zoetis (5 mg/kg) was delivered intramuscularly as a systemic antibiotic on the day of surgery and 7 days post-surgery. Wounds were monitored and routinely evaluated by veterinary staff. The pigs were euthanized 13 days post-surgery via intravenous injection of euthasol (130 mg/kg), and wound samples were collected for histology.
Anaesthesia:	Adolescent female Yorkshire pigs were anesthetized with a cocktail of telazol (4.4 mg/kg), ketamine (2.2 mg/kg), and xylazine (2.2 mg/kg) administered intramuscularly and maintained under isoflurane for the duration of the surgery.
Analgesia:	Bupranex (0.01 mg/kg) was administered as a single injection prior to surgery as a fast-acting analgesic followed by the application of a transdermal fentanyl patch (50 mcg/hr) for sustained analgesia (72 hours).
Locations:	S.R. Light Laboratory Surgical Facility
Acclimatisation period:	The pigs are received from a commercial supplier and will undergo a minimum of four days of acclimationbefore procedures are performed.

Animal care and monitoring

Adverse events:	Potential adverse events include wound/skin infection and inflammatory reactions to test compounds.
Humane endpoints:	If the infection appears to be involving the bulk of the wounds or the sides and ventral surface and/ordoes not improve with additional antibiotics then the animal will be euthanized. We will include a veterinarian on this decision and abide by their recommendation. If additional medication fails to control pain or itching and there is extreme damage to multiple wounds by scratching, the pig will be euthanized. We will include veterinarian on this decision and abide by the recommendation.

Welfare monitoring:	Animals were checked twice daily for 3 days after surgery and then once daily during the work week (M-F) until the end of the experiment. Of course, if there seems to be a concern, then the animal was monitored several times a day including weekends. If minor infection occurs, antibiotics were continued. We monitored daily such things as inability to ambulate, self-trauma, inability/failure to eat or drink,and hunched posture. During dressing changes we look for deepening ulceration within the wound bed and severe skin or wound infections. During dressing changes we look for redness of skin, increased blood flow, excessive warmth, ulceration at the wound site. There can be behavioral manifestations of pain such as hyperactivity and frenzied motions to excessively scratch at the wounds by rubbingagainst the cage. Veterinarian was consulted to suggest possibility of additional drugs (i.e. antihistamine for itching)	Attached clinical assessment form:	N
Changes in housing and husbandry :	Pigs were housed singly upon arrival and throughout the study to prevent damage to the normal unwounded skin pre-surgery and then wound dressings post-surgery by other pigs. This also allows for reduced interference with the wounds and/or adjunctive bandaging system during the recovery phase of anesthesia. The pigs had visual, olfactory and touch contact with neighboring pigs if any were in house. We adhere to DAC's Environmental enrichment programs for our pigs including extra environmental enrichment per DAC's Isolated Animal Procedures if no other animals are in house while our pig is present.		
Restrictions in veterinary care:	Pigs were fasted for a minimum of 12 hours and a maximum of 18 hours prior to administration of anathesia to prevent aspiration.		

Risks

Emergency procedures:	N/A
Potential risk to personnel:	Risks to personel were minimized through safe handling of biologic test substances. We expected minimal to no toxicity with the use of either the biologics or Pluronic 127. Yogev et al (2010) have demonstrated that application of Pluronic 127 gel topically in mice showed no significant differences in terms of inflammation compared to DI water (control).

Personnel involved in the experiment

Mariah Bezold	NSP Dressing Change, NSP: Imaging, SS Excisional Wound Creation Surgery, NSP Treatment Application	Trained and competent☒
Josh McCune	NSP Dressing Change, NSP: Imaging, SS Excisional Wound Creation Surgery, NSP Treatment Application	Trained and competent☒

Study design and sample size

Experimental groups:	To mitigate problems associated with the slow uptake of rHDL liquid formulation by the wounds in pigs and improve drug delivery, this study evaluated the Pluronic F-127 thermosensitive hydrogel for topical delivery of rHDL (1:66 PC). The study was focused on the optimization of the delivery regimen, specifically on selection of the appropriate drug concentration and drug volume applied to wounds at pre-defined dose of rHDL. The effects of topically applied rHDL with respect to healing rates, wound perfusion and wound re-epithelialization were investigated. Study was performed in non-ischemic, full-thickness, excisional skin wound model in two pigs to guide future efficacy studies in porcine ischemic and diabetic wound healing models. Wounds of each pig were treated with rHDL reconstituted in 20% pluronic gel at various doses and volumes (0.15 mg in 75 µL, 0.3 mg in 75 µL, 0.3 mg in 150 µL, and 0.6 mg in 150 µL) or 20% pluronic gel vehicle (75 µL or 150 µL).
Experimental unit:	Wound — Sample size per group: N=8
Justification for sample size:	For wound studies with active biologics formulated in Pluronic 127, we needed to study up to 6 treatments with N=8 to be able to reject the null hypotheses that the mean of the experimental and control groups are equal with probability (power)0.9. The Type I error probability associated with this test of this null hypothesis is 0.01. We would need a total of 2 pigs for the study.
EDA read only diagram:	Link to EDA read only diagram — Access code: Enter

Inclusion and exclusion criteria

Inclusion criteria:	All the animals were purchased from Oakhill Genetics. The animals were requested to be shipped between 27-36 kgs and 16-18 weeks of age. The animals were acclimatized for 3-5 days prior to surgery. Animals were included in the study.
Exclusion criteria:	Any animal that experiences any of the humane endpoints described above will be euthanized and excluded from the study. Any animal that contracts infection will receive antibiotics as described above and all infected wounds will be excluded from the study.
Expected attrition:	The expected attrition of animals when performing non-ischemic excisional wound healing studies is 10%. The attitrion observed in this non-ischemic excisional wound healing studies is 0% since all animals survived.

Randomisation and blinding/masking	
Method of allocation to group:	All treatment groups were randomized across varied positions along the dorsal region of the animals to account for positional and anatomical variability in this model.
Strategy to minimise confounders:	All treatment groups were randomized across varied positions along the dorsal region of the animals to account for positional and anatomical variability in this model. All treatment groups were randomly placed in equal numbers across four quandrants of the dorsal region of the animals including equal numbers on the right cephalic region, the left cephalic region, the right caudal region, and the left caudal region.
Blinding strategy:	All treatment groups were randomized across varied positions along the dorsal region of the animals to account for positional anatomical variability in this model. All measurements were blinded to personnel in the study for treatment and analysis.

Outcome measures and statistical methods

Outcome measures:	Wound closure measurements: Wound area was measured using photographs of each individual wound in the same plane as a sterile ruler placed directly adjacent to the wound. Wound area was determined quantitatively through calibration with ruler in each macroscopic photograph, and wound size was calculated as a percentage of wound area measured on POD 0 using ImageJ. Laser Doppler perfusion imaging: A laser doppler imager (moorLDI2-HIR; Moor Instruments) was used to track blood perfusion within the wounds over the duration of the study. Flux images of wounds were obtained before the application of therapeutics (this was done to avoid interference from the treatment) during dressing changes at days 3, 6, 8, 10, and 13 post-surgery. Blood perfusion within a 2 x 1 cm region of interest was quantified using Moor analysis software. Data are presented relative to both normal unwounded skin and gel-treated wounds. Histology (H&E, Trichrome): Tissue samples were fixed in 10% neutral buffered formalin for 48 hours and were then dehydrated in a graded ethanol series, exposed to xylene, and embedded in paraffin. Tissue sections (5µm thick) were deparaffinized in gradients of xylene and ethanol and rehydrated in Tris-Buffered Saline/0.1% Tween 20 (TBST) buffer. Gomori's Trichrome staining and hematoxylin and eosin (H&E) staining were performed according to the manufacturer's recommendation. All slides were scanned at 20X by Leica SCN400 Brightfield Slide Scanner for whole slide imaging and further analysis by ImageJ. Immunohistochemistry: Tissue samples were fixed in 10% neutral buffered formalin for 48 hours and were then dehydrated in a graded ethanol series, exposed to xylene, and embedded in paraffin. Tissue sections (5µm thick) were deparaffinized in gradients of xylene and ethanol and rehydrated in Tris-Buffered Saline/0.1% Tween 20 (TBST) buffer. Antigen retrieval was performed using citrate-based pH 6 antigen retrieval solution (Dako) for 1 minute at 110°C and allowed to cool to 90°C in a decloaking chamber. Sections were then incubated for 40 mins in 3% H2O2 TBST solution and blocked with protein block (Dako) for 20 mins. Sections were further incubated with mouse anti-cytokeratin14 antibody for 60 mins at room temperature. Secondary antibodies donkey anti-mouse HRP were applied for 30 mins at RT and samples were exposed DAB substrate (Dako) for 5 mins. Slides were then rinsed in TBST buffer, dehydrated in graded ethanol and xylene solutions, and mounted with Acrytol mounting media. All slides were scanned at 20X by Leica SCN400 Brightfield Slide Scanner for whole slide imaging and further analysis by ImageJ.

Primary outcome measure:	Quantification of wound re-epithelization was performed with sections immunohistologically stained for Cytokeratin 14, a marker of epithelial cells, as well as with sections histologically stained by H&E. Quantification of wound re-epithelization by Cytokeratin 14 was conducted on blinded immunohistochemical sections which were evaluated by ImageJ Software to quantify the percentage closure of epithelia layer by positive nuclear staining for Cytokeratin 14 across the wound bed.
Analysis plans:	Data was analysed via one-way analyses of variance (ANOVA) followed by Tukey's pairwise comparison was performed using GraphPad Prism to define differences between treatments at a given time point.

Sign off			
Primary responsible:	N/A	Date	☐ I confirm I am aware of my responsibilities as the primary responsible (e.g. conditions of the personal licence) and my training and competency record is up to date.
Project lead:	N/A	Date	☐ I confirm I am aware of my responsibilities as a project lead/licence holder and this work is in line with the project.
Internal sign off:	N/A	Date	Role, e.g. NACWO
