# Supplementary material for: Evaluation of Topical Reconstituted HDL as a Treatment for Diabetic Wounds in Murine and Porcine Models
Source: Biomolecules. 2026 Jul 9;16(7):1001. doi: 10.3390/biom16071001 (PMC13407075; doi:10.3390/biom16071001)

20220307

Stability of rHDL in Pluronic gel (20%) in PBS or DMEM

rHDL at 0.5, 1, 2 or 4 mg/ml added to 20% (W/V) Pluronic F-127 prepared on PBS or DMEM at 4°C.
Samples of rHDL in Pluronic gel separated with Native PAGE and stained with Coomassie Blue. The PAGE was run at 4°C
5 μg of rHDL loaded per line.

A. **Pluronic gel in PBS**
B. **Pluronic gel in DMEM**

Coomassie Blue

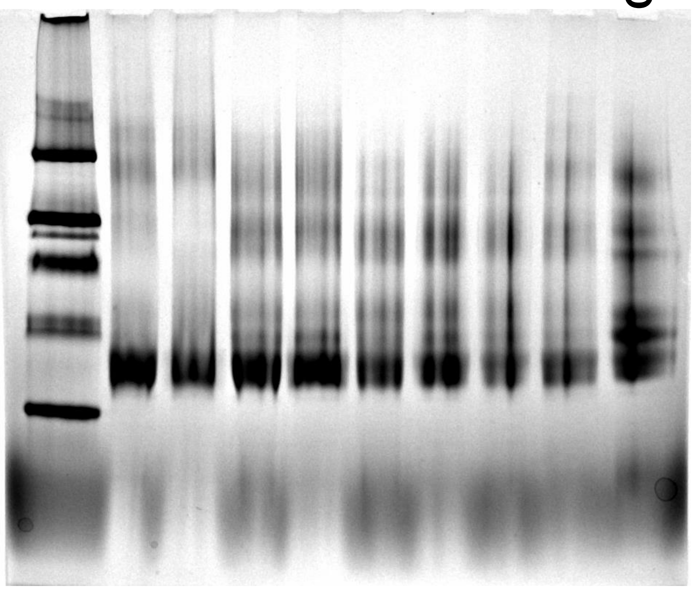

Supplement: Supplementary file 1 [file biomolecules-16-01001-s001.zip › biomolecules-4343985-gel.pdf]
