# Supplementary material for: Evaluation of Topical Reconstituted HDL as a Treatment for Diabetic Wounds in Murine and Porcine Models
Source: Biomolecules. 2026 Jul 9;16(7):1001. doi: 10.3390/biom16071001 (PMC13407075; doi:10.3390/biom16071001)
Supplement: Supplementary file 1 [file biomolecules-16-01001-s001.zip › Wound-Healing rHDL_Supplemental_Material.pdf]

Supplemental Material

Evaluation of topical reconstituted HDL as a treatment for diabetic wounds in murine and porcine models

Juan E. Camacho Londoño[1,*], Sharelle Sturgeon[2,†], Yun Dai[2], Alexey Navdaev[3,†], Padmapriya Ponnuswamy[1], Brandon H. Greene[1,†], Craig L. Duvall[4], Prarthana Patil[4], Joshua McCune[4], Mariah Bezold[4], Anil Dolgun[2], Justin R. Hamilton [2], Bronwyn A. Kingwell[2], Alberto B. Silva[3], Svetlana Didichenko[3,†], Helen Cao [2]

[1]CSL Innovation GmbH, Emil-von-Behring-Straße 76. 35041 Marburg, Germany
[2]CSL Innovation, 655 Elizabeth Street, Melbourne, VIC 3000, Australia
[3]CSL AG, Tellstrasse 18, 3014 Bern, Switzerland
[4]Department of Biomedical Engineering, Vanderbilt University, Nashville, TN 37240, USA.
*Correspondence: juan.camacholondono@cslbehring.com or juan.londono@pharma.uni-heidelberg.de;
[†]Authors were employees of CSL when the study was conducted

Supplemental Methods

Studies in diabetic mice.

Mice were anesthetized with a single injection of ketamine (100 mg/kg)/xylazine (20 mg/kg) Lyppard, Australia). Two 6-mm circular full-thickness excisional wounds were surgically created on either side of the dorsal skin after depilation. A donut-shaped 12-mm silicon splint (Thermo Fisher) was then placed around the wounds and adhered to the skin with superglue (Bostik) and interrupted 6-0 nylon sutures (Dynek). The wounds were covered with a transparent occlusive dressing (OpSite, Smith & Nephew). Following the surgery, the mice were housed in individual cages and were subcutaneously administered carprofen (4.4 mg/kg, Lyppard Australia) once daily for post-operative pain relief.

For histological and immunohistochemical analysis, the wound tissues were fixed in 10% neutral buffered formalin (ThermoFisher Scientific) for 24 hours, embedded in paraffin and sectioned at 4 μm. Collagen was visualized by Masson's trichrome staining (fast green). For neutrophil detection, the sections were incubated with the primary antibody against Ly6G/C (Clone NIMP-R14, Abcam, 1:100), then incubated with the VisUCyte HRP-conjugated goat anti-rat secondary antibody (R&D Systems, 1:2). For macrophage detection and alpha smooth muscle actin (αSMA) staining, the sections

were incubated with the rabbit polyclonal antibodies against CD68 (Abcam, 1:1000) and αSMA (Abcam, 1:500), respectively. The sections were then incubated with the HRP-conjugated Dako EnVision+ System goat anti-rabbit secondary antibody (Agilent, Ready-to-use). The primary and secondary antibodies were incubated at room temperature for 1 hour and 30 min, respectively. The slides were scanned using a Vectra Polaris imaging system and the positive staining was quantified using Qupath software.

Pilot studies in pigs.

For wound studies using active biologics formulated in Pluronic F127, a study design including up to six treatments with N=8 wounds per treatment group was estimated to provide 90% power to reject the null hypothesis that the means of the experimental and control groups were equal. The Type I error probability associated with this test was set at 0.01. Based on this design, a total of two pigs were required for the study. Adolescent female Yorkshire pigs were anesthetized with a cocktail of telazol (4.4 mg/kg), ketamine (2.2 mg/kg), and xylazine (2.2 mg/kg) administered intramuscularly (i.m.) and maintained under isoflurane for the duration of the surgery. Bupranex (0.01 mg/kg) was administered as a single injection prior to surgery as a fast-acting analgesic followed by the application of a transdermal fentanyl patch (50 mcg/h) for sustained analgesia (72 hours). Dorsal skin was shaved and disinfected using 70% ethanol, chlorohexidine 2% scrub, chlorohexidine 2% solution, and betadine washes.

Scalpel blades were used to cut wounds to a full thickness depth in the dorsal region of each pig. The epidermis, dermis, and underlying fat were removed to expose the fascia layer below. The depths of the wounds were measured at approximately 5–6 mm. Wounds were covered with layering's of Mepilex Transfer (Molnlycke Healthcare), Mextra absorbent pad (Molnlycke), OpSite adhesive (Smith&Nephew), MediChoice Tubular Net Bandage (Owens & Minor), and Vetwrap (3M) bandaging. The dressings were changed every other day.

Wounds were treated on post-operation day (POD) 1, 3, 6, 8, and 10. Wounds were cleaned with chlorohexidine 2% solution and saline, and then were allowed to dry prior to the application of treatments by positive displacement pipette. Excede® (Zoetis) 5 mg/kg was delivered i.m. as a systemic antibiotic on the day of surgery and 7 days post-surgery. Wounds were monitored and routinely evaluated by veterinary staff. The pigs

were euthanized 13 days post-surgery via intravenous (i.v.) injection of euthasol (130 mg/kg), and wound samples were collected for histology. Tested articles were prepared by CSL Behring and thawed prior to application.

The wound area was measured using photographs of each individual wound in the same plane as a sterile ruler placed directly adjacent to the wound; the area was determined quantitatively through calibration with ruler in each macroscopic photograph. Wound size was calculated as a percentage of wound area measured on POD 0 using ImageJ software.[32] Tissue samples were fixed in 10% neutral buffered formalin for 48 hours and were then dehydrated in a graded ethanol series, exposed to xylene, and embedded in paraffin. Tissue sections (5 µm thick) were deparaffinized in gradients of xylene and ethanol and rehydrated in Tris-Buffered Saline/0.1% Tween 20 (TBST) buffer. Gomori's Trichrome staining and hematoxylin and eosin (H&E) staining were performed according to the manufacturer's recommendation. For immunohistochemistry (IHC), antigen retrieval was performed using citrate-based pH 6 antigen retrieval solution (Dako) for 1 min at 110°C and allowed to cool to 90°C in a decloaking chamber. Sections were then incubated for 40 mins in 3% H_2O_2 TBST solution and blocked with protein block (Dako) for 20 mins. Sections were further incubated with mouse anti-cytokeratin-14 antibody (Clone LL002 BioRad, 1:3400) for 60 mins at room temperature. Secondary donkey anti-mouse HRP antibodies were applied for 30 mins at room temperature and samples were exposed with DAB substrate (Dako) for 5 mins. Slides were then rinsed in TBST buffer, dehydrated in graded ethanol and xylene solutions, and mounted with Acrytol mounting media (Leica Biosystems). All slides were scanned at 20× by Leica SCN400 Brightfield Slide Scanner for whole slide imaging and further analyzed by ImageJ. Finally, data was plotted as arithmetic mean ± standard deviation (SD). One-way analyses of variance (ANOVA) followed by Tukey's pairwise comparison was performed using GraphPad Prism software to define differences between treatments at a given time point.

Studies in diabetic Yucatan miniature pigs

After approximately 2 months of confirmed diabetes induction and at an age between 8–12 months (25–50 kg) animals were anesthetized with Tiletamine/zolazepam (2–6 mg/kg, i.m.), xylazine (0.4–1.2mg/kg, i.m.) and atropine (0.04–0.05 mg/kg, i.m.) to produce the wounds. Animals undergoing anesthesia for surgery procedures, dose administration,

and dressing changes were food fasted for at least 8 hours. Anesthesia was induced and/or maintained with isoflurane. For analgesia buprenorphine ER (extended release) (0.2–0.3 mg/kg, s.c.) and carprofen (2.2–4.4 mg/kg i.m.) were included. Cefazolin (20 mg/kg, i.v.) and Excede® (5 mg/kg, i.m.) were administered at the time of procedure for prophylaxis. A surgical scrub of each animal's skin was performed prior to wound creation. Full-thickness excisional wounds were made using an aseptic surgical technique. The wound sites were positioned along a paraspinal column on each side, ensuring the column remained between the crest of the shoulders and the ilium. Each animal had eight wound sites (one row of four sites per side) with a diameter of 2.5 cm (~5 cm^2), and appropriate depth (full thickness) spaced at least 3 cm apart (Figure S2). Following creation of wounds, the wound bed was cleaned with sterile saline and/or gauze to remove any foreign matter or loose tissue debris. Clinical blood parameters were monitored during the acclimation (prior to randomization) and prior to termination.

Dose administration, dermal scoring, and wound planimetry in diabetic pigs. Vehicle, rHDL, or albumin control were applied directly to the designated wound site(s) daily, from dosing phase day 1 for up to 18 days (or when ~70–80% wound closure was achieved in any of the groups with the fastest healing). An appropriate sterile pipette tip was used to add 0.5 mL of the formulation directly to the middle of the designated wound bed to cover it completely (without spreading over) and allowed to solidify (~1–2 mins). Becaplermin was applied at 0.3 g (equivalent to 1.2 cm from a 15 g tube) as recommended by the manufacturer. Each animal received each treatment in at least one wound (Figure S2). Each wound site was then covered with a barrier dressing of non-adherent sterile gauze and transparent film. Once all sites were dosed and barrier dressing applied, the entire wound area was covered with a layer of foam pad and tear-resistant mesh (or stockinette) to prevent dressing materials from moving. Wounds were scored using a modified Bates-Jensen dermal scoring prior to dosing on days 1, 5, 8, 12, 14, 17 and 18 of the dosing phase and features such as wound edge, exudate type and amount, granulation tissue and epithelialization were considered. On the same days, planimetry photography was performed prior to dose application using a SilhouetteStar ARANZ Medical camera. Each image was taken directly in front of the wound to ensure an accurate measurement and included colored ruler, and appropriate identifiers (study

number, animal ID, site number, and study day/date of collection). Image analysis was performed using SilhouetteConnect software to calculate change in wound area with respect to dosing phase day. On day 18, tissue samples from the wounds were collected during necropsy, fixed in 10% neutral buffered formalin and processed to obtain paraffin blocks and histological slides for histopathological evaluation. A complete gross necropsy was performed on all study animals, and the wound sites were excised with a 0.2 cm margin of surrounding normal skin (peri-wound area, Figure S2).

Note that in the diabetic mouse studies, each wound measured 28.26 mm^2. The doses applied were 75, 50, 30, and 10 µg per wound, corresponding to 2.65, 1.77, 1.06, and 0.35 µg/mm^2, respectively. In the diabetic miniature pig studies, each wound measured 490 mm^2. rHDL was formulated at 10, 5, and 2 mg/mL, with an application volume of 0.5 mL per wound, resulting in doses of 5, 2.5, and 1 mg per wound, equivalent to 10.2, 5.1, and 2.04 µg/mm^2, respectively. Thus, the lowest dose in the diabetic miniature pig study (2.04 µg/mm^2) is comparable to the highest doses used in mice (2.65 and 1.77 µg/mm^2), ensuring translational relevance across species

Histopathological analysis of samples from diabetic pigs

Two independent pathologists (at StageBio, US and Anapath, Switzerland), analyzed tissue sections from each wound site. The wound sites were cut in half and the halves that contained peri-wound areas (Figure S2) were histologically processed. The sections were stained with H&E to assess general morphology and Herovici to assess collagen maturity. All slides were then shipped to StageBio for pathological evaluation. The slides were evaluated according to the ISO 109931 part 6, Annex E standard, with additional assessment of wound-bed specific features. The slides were evaluated for inflammation and inflammatory cell types, wound bed healing, tissue response and reactivity score (ISO 10993:6). Additional tissue sections were generated and analyzed at AnaPath, using histomorphometry by image analysis and scoring defined by the ISO 10993-6:2016 (Table 1). Briefly, paraffin blocks were sectioned and stained with H&E and Mason's Trichrome (MT) (one slide per block for each stain). The H&E and MT-stained slides from all wound sites were Whole Slide Imaged (WSI) scanned by an Olympus Slideview VS200 slide scanner using a VS-264C camera and the 20× objective. Quantitative evaluation was performed on the MT WSI using Olympus imaging and image analysis software cellSens

v3.1. Diagnostic criteria and terminology used throughout the study were based on recognized texts, current scientific literature as well as Registry of Industrial Toxicology Animal-data (RITA), Society of Toxicologic Pathology (STP) and the International Harmonization of Nomenclature and Diagnostic (INHAND) terminology.

Table S1. Summary of hematology, serum chemistry and coagulation parameters

Parameter	Before wound creation (d-3)		End observation period (d18)	
	Mean	SD	Mean	SD
**Hematology**				
**White blood cell count (×10³ cells/µL)**	10.10	2.43	10.03	1.05
**Red blood cell count (×10⁶ cells/µL)**	6.37	0.90	6.87	0.45
**Hemoglobin (g/dL)**	12.33	1.64	13.48	0.94
**Hematocrit (%)**	36.48	4.84	40.41	2.80
**Mean corpuscular volume (fL)**	57.38	2.23	58.86	2.67
**Mean corpuscular hemoglobin (pg)**	19.38	0.62	19.59	0.76
**Mean corpuscular hemoglobin concentration (g/dL)**	33.78	0.39	33.30	0.48
**Platelet count (×10³ cells/µL)**	541.13	114.50	541.63	63.04
**Neutrophil count (×10³ cells/µL)**	4.13	1.89	3.61	0.93
**Neutrophils (%)**	39.43	8.71	35.70	7.31
**Lymphocyte count (×10³ cells/µL)**	5.14	0.98	5.53	0.75
**Lymphocytes (%)**	52.08	7.89	55.54	7.54
**Monocyte count (×10³ cells/µL)**	0.44	0.17	0.50	0.05
**Monocytes (%)**	4.38	1.66	4.98	0.44
**Eosinophil count (×10³ cells/µL)**	0.27	0.20	0.25	0.11
**Eosinophils (%)**	2.86	1.95	2.51	0.98
**Basophil count (×10³ cells/µL)**	0.03	0.01	0.03	0.01
**Basophil (%)**	0.29	0.14	0.31	0.11
**Reticulocyte count (×10⁹ cells/L)**	38.60	19.78	45.43	20.54
**Reticulocytes (%)**	0.59	0.29	0.65	0.27
**Serum chemistry**				
**Albumin (g/dL)**	3.41	0.28	3.31	0.30
**Alkaline phosphatase (U/L)**	111.50	17.94	112.38	17.58
**Alanine aminotransferase (U/L)**	54.13	7.01	48.25	8.15
**Amylase (U/L)**	914.25	162.17	874.88	150.76
**Aspartate aminotransferase (U/L)**	33.25	12.90	20.75	6.89

**Direct bilirubin (mg/dL)**	0.01	0.03	0.00	0.00
**Total bilirubin (mg/dL)**	0.18	0.04	0.19	0.03
**Calcium (mg/dL)**	10.03	0.23	9.58	0.16
**Cholesterol (mg/dL)**	115.00	10.54	120.63	9.33
**Creatine kinase (U/L)**	212.86 *	54.29	325.38	409.18
**Creatinine (mg/dL)**	0.81	0.09	0.83	0.10
**Gamma glutamyltransferase (U/L)**	53.75	8.12	59.38	8.76
**Glucose (mg/dL)**	252.88	72.40	365.38	31.55
**Lactate dehydrogenase (U/L)**	412.00	86.82	399.00	56.26
**Inorganic phosphorus (mg/dL)**	6.26	0.47	6.41	0.26
**Total protein (g/dL)**	6.54	0.41	6.65	0.43
**Triglycerides (mg/dL)**	26.50	5.72	56.00	27.29
**Blood urea nitrogen (mg/dL)**	19.38	2.34	19.00	1.00
**Sodium (mEq/L)**	137.00	2.45	135.38	2.12
**Potassium (mEq/L)**	4.14	0.21	4.68	0.36
**Chloride (mEq/L)**	98.38	2.29	96.88	2.09
**Globulin (g/dL)**	3.13	0.47	3.35	0.49
**Albumin / globulin ratio**	1.11	0.20	1.03	0.23
**Coagulation**				
**Prothrombin time (s)**	14.59	0.33	14.40	0.37
**Activated partial thromboplastin time (s)**	14.80	1.37	13.84	1.50

Mean value for 8 animals at each sampling point for all parameters except for creatinine kinase(*). Sorbitol dehydrogenase was determined but results were not included because the levels were below the level of quantification.
SD, standard deviation; s, seconds

Table S2. Wound area reduction on Day 18 (differences in mean response with 95% CI).

Parameter	Estimate	95% CI	p-value
rHDL vs vehicle			
2 mg/mL	−0.52	(−9.63, 8.58)	0.998
5 mg/mL	−2.04	(−10.92, 6.83)	0.909
10 mg/mL	3.44	(−5.46, 12.33)	0.691
rHDL vs becaplermin			
2 mg/mL	−9.13	(−18.25, −0.02)	0.049*
5 mg/mL	−10.65	(−19.55, −1.75)	0.014*
10 mg/mL	−5.17	(−14.06, 3.71)	0.380
rHDL vs albumin			
2 mg/mL	2.64	(−6.65, 11.93)	0.842
5 mg/mL	1.12	(−7.96, 10.20)	0.983
10 mg/mL	6.60	(−2.49, 15.69)	0.210

CI, confidence interval; rHDL, reconstituted high-density lipoprotein.

Table S3. Summary of local effects/host reaction based on the average full score.

Treatment	Vehicle (PBS)	Becaplermin	rHDL 10 mg/mL	rHDL 2 mg/mL	Albumin 10 mg/mL	rHDL 5 mg/mL
**Vehicle (PBS)**	-	2.0	−0.9	−1.2	−1.4	−4.0
**Becaplermin**	−2.0	-	−2.9	−3.2	−3.4	−6.0
**rHDL 10 mg/mL**	0.9	2.9	-	−0.3	−0.5	−3.1
**rHDL 2 mg/mL**	1.2	3.2	0.3	-	−0.2	−2.8
**Albumin 10 mg/mL**	1.4	3.4	0.5	0.2	-	−2.6
**rHDL 5 mg/mL**	4.0	6.0	3.1	2.8	2.6	-

PBS, phosphate-buffered saline; rHDL, reconstituted high-density lipoprotein.

Table S4. Summary of ISO 10993-6 scores across the different treatment groups.

Groups	Vehicle (PBS)	Becaplermin	Albumin 10 mg/mL	rHDL 2 mg/mL	rHDL 5 mg/mL	rHDL 10 mg/mL
**Epidermis**						
Polymorphonuclear cells	0.0	0.0	0.0	0.0	0.0	0.1
Erosion/ ulceration	2.0	1.7	2.5	2.8	2.5	2.0
Subtotal x 2	4.0	3.5	5.0	5.6	5.0	4.2
**Total Epidermis**	4.0	3.5	5.0	5.6	5.0	4.2
**Dermis**						
Polymorphonuclear cells	1.1	1.1	1.2	1.0	1.3	1.1
Lymphocytes	1.0	1.0	1.1	1.1	1.2	1.1
Plasma cells	0.7	0.4	0.3	0.5	0.4	0.5
Macrophages	0.0	0.2	0.5	0.2	0.5	0.2
Giant cells	0.1	0.2	0.6	0.4	0.7	0.4
Necrosis	1.2	0.5	1.0	0.9	1.1	1.0
Subtotal x 2	8.2	6.5	9.4	8.2	10.4	8.5
Mineralization	0.1	0.0	0.0	0.1	0.0	0.0
Hemosiderin	0.4	0.	0.6	0.4	0.6	0.5
Hemorrhage/edema	0.5	0.5	0.3	0.4	0.3	0.5
Subtotal	1.0	1.1	0.9	0.9	0.9	1.1
**Total Dermis**	9.2	7.6	10.3	9.1	11.3	9.6
**Subcutis**						
Polymorphonuclear cells	0.1	0.2	0.2	0.2	0.1	0.3
Lymphocytes	1.0	0.9	1.0	1.0	1.0	0.9
Plasma cells	0.4	0.0	0.3	0.1	0.5	0.3
Macrophages	0.1	0.2	0.0	0.1	0.1	0.1
Giant cells	0.3	0.6	0.1	0.3	0.5	0.4
Necrosis	0.0	0.0	0.0	0.0	0.0	0.0
Subtotal x 2	3.6	3.8	3.2	3.4	4.4	3.8
Hemosiderin	0.5	0.5	0.3	0.5	0.7	0.6
Mineralization	0.0	0.0	0.0	0.0	0.0	0.0
**Total Subcutis**	4.2	4.3	3.5	3.9	5.1	4.5
**Average FULL Score**	17.4	15.4	18.8	18.6	21.4	18.3

PBS, phosphate-buffered saline; rHDL, reconstituted high-density lipoprotein.

Table S5. Histological cutaneous parameters for the assessment of wound healing performance.

Parameters to evaluate	Score				
	0	1	2	3	4
**Re-epithelialization**	0	Epidermis covering <25% of the surface	Epidermis covering 25-50% of the surface	Epidermis covering 50-75% of the surface	Epidermis covering >75% of the surface
**Granulation tissue (defect tissue replacement)**	0	Tissue replacing <25% of the defect	Tissue replacing 25%-50% of the defect	Tissue replacing approx. > 50-75% of the defect	Tissue replacing more than 75 % of the defect
**Collagen Maturation***	0	Most of collagen fibers – vertical, with reticular pattern, and reactive fibroblasts	Mixed of horizontal and vertical collagen fibers, with mixed reticular/fascicular pattern, and reactive fibroblasts	Predominance of horizontal collagen fibers, with predominance of fascicular pattern, and reactive fibroblasts	Mature collagenous matrix, with horizontal fascicles of collagen fibers, and fibrocytes
**Serocellular crust**	0	Covering <25% of defect	Covering 25-50% of defect	Covering 50-75% of defect	Covering >75% of defect

* Evaluated on Masson Trichrome stained slides.

Table S6. Summary of Hodges-Lehman effect estimates and p-values corresponding to Table 1.

Treatment	Becaplermin	Albumin 10 mg/mL	rHDL 10 mg/mL	rHDL 5 mg/mL	rHDL 2 mg/mL
	HL (p-value)	HL (p-value)	HL (p-value)	HL (p-value)	HL (p-value)
**Re-epithelialization**	0.00 (0.852)	-0.75 (0.285)	-0.25 (0.852)	-0.50 (0.285)	-1.00 (0.285)
**Granulation tissue (defect tissue replacement)**	0.00 (1.000)	0.00 (1.000)	0.00 (1.000)	0.00 (1.000)	0.00 (1.000)
**Collagen maturation**	0.00 (1.000)	0.00 (1.000)	0.00 (1.000)	0.00 (1.000)	0.00 (1.000)
**Serocellular crust**	0.50 (0.359)	0.50 (0.599)	0.25 (0.654)	0.50 (0.432)	0.25 (0.654)

Hodges-Lehmann (HL) effect size estimates of score shift and p-values from HL aligned ranks test adjusted by Bonferroni-Holm procedure for comparison of all active treatments against vehicle control PBS, phosphate-buffered saline; rHDL, reconstituted high-density lipoprotein.

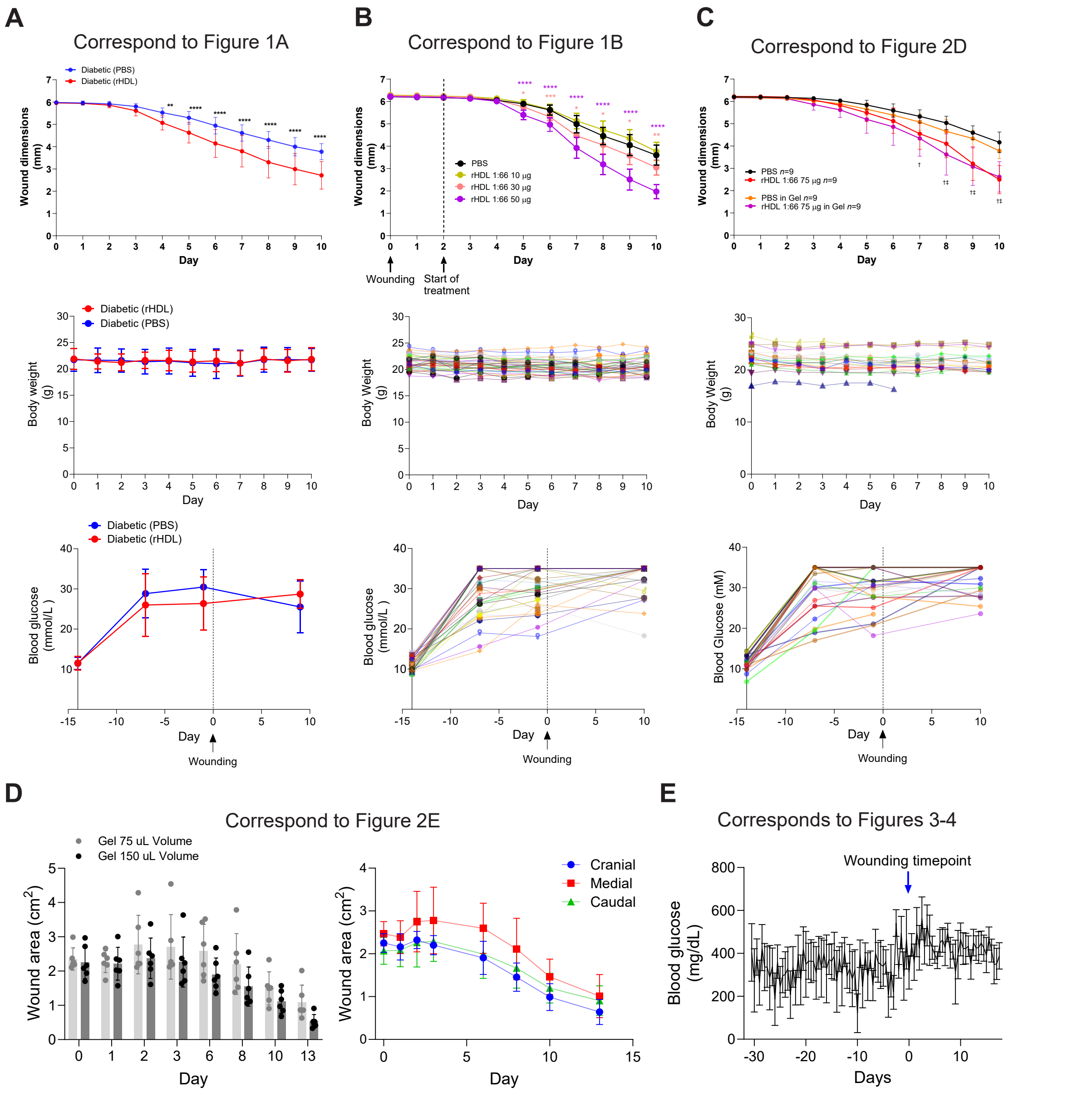

Figure S1: Absolute wound size, body weight and blood glucose. Wound closure, body weight and blood glucose from mice corresponding to the analyses presented in **(A)** Figure 1A, **(B)** Figure 1B and **(C)** Figure 2D. Mean values (± standard deviation) for body weight and blood glucose are shown in **(A)** as both wounds on each animal received the same treatment, in contrast to **(B)** and **(C)** where each animal received different treatments on each wound, and therefore the individual body weight and glucose levels are shown for every mouse. In **(C)** the body weight of one animal is depicted until day 6 because this animal was taken out from the study before the end of the observation period. In **(A)** and **(B)** * $p<0.05$, ** $p<0.01$, *** $p<0.001$, **** $p<0.0001$ represent comparisons with the PBS group. In **(C)** † denotes significance ($p<0.05$) between rHDL (1:66) 75 µg in PBS and PBS alone; ‡denotes significance ($p<0.05$) between 75 µg rHDL in 20% Pluronic F-127 gel (1:66) 75 µg and 20% Pluronic F-127 (PBS in gel). **(D)** Absolute wound area corresponding to Figure 2E where two volumes of 20% Pluronic F-127 Pluronic gel applied to wounds in healthy pigs, in terms of wound closure, were compared. **(E)** Blood glucose levels determined twice a day before and during the study in diabetic minipigs (n=8). Data are presented as mean ± standard deviation.

A

Pilot experiment (test of gel formation)

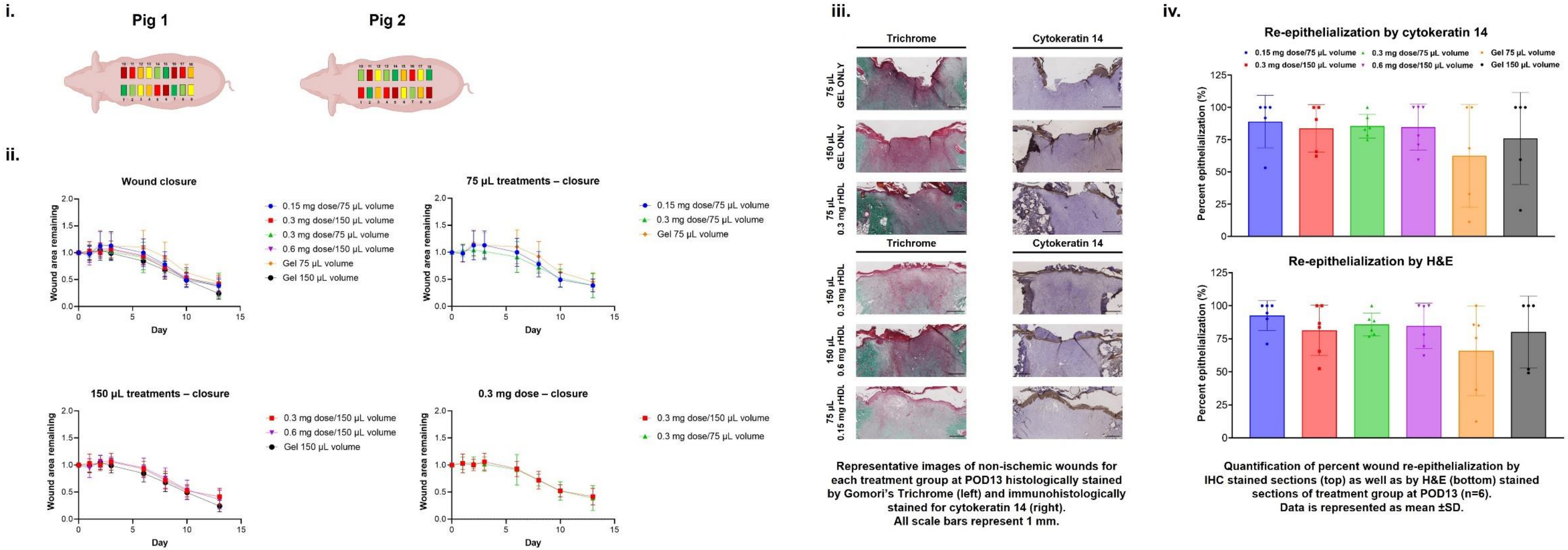

Representative images of non-ischemic wounds for each treatment group at POD13 histologically stained by Gomori's Trichrome (left) and immunohistologically stained for cytokeratin 14 (right). All scale bars represent 1 mm.

Quantification of percent wound re-epithelialization by IHC stained sections (top) as well as by H&E (bottom) stained sections of treatment group at POD13 (n=6). Data is represented as mean ±SD.

B

Experimental design and sampling strategy

i.

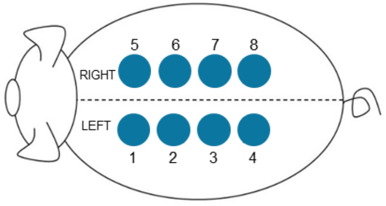

Allocation of dose site

	Letter code of Test Material	Test Material
Blue	A	Vehicle
White	B	Becaplermin
Orange	C	rHDL 10 mg/mL
Pink	D	rHDL 2 mg/mL
Green	E	Albumin
Grey	F	rHDL 5 mg/mL

Animal ID	Site 1 (L1)	Site 2 (L2)	Site 3 (L3)	Site 4 (L4)	Site 5 (R5)	Site 6 (R6)	Site 7 (R7)	Site 8 (R8)
1001	A	B	C	D	E	F	A	B
2001	C	D	E	F	A	B	C	D
3001	E	F	A	B	C	D	E	F
4001	F	A	B	C	D	E	F	A
5001	B	C	D	E	F	A	B	C
6001	D	E	F	A	B	C	D	E
7001	F	A	B	C	D	E	F	A
8001	B	C	D	E	F	A	B	C

L, left; R, right.

ii.

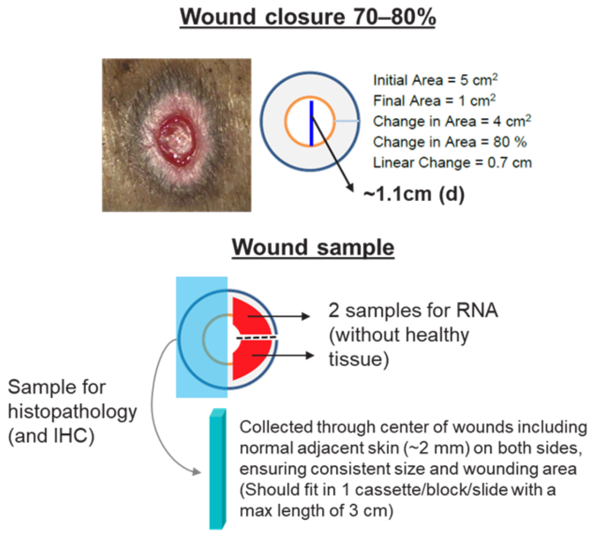

Figure S2: ***In vivo*** **characterization of the rHDL gel formulation suitable for studies in pigs. (A) Pilot experiment (evaluation of the gel formulation). i.** Wounds (18/pig; 2 × 1 cm) were created in two rows of nine, approximately 3 cm from the spine. **ii.** Combined wound closure rates of all treatments, 75 µL treatments, 150 µL treatments, and 0.3 mg doses in pigs 1 and 2. Treatments: rHDL in 20% Pluronic F-127 at varying doses and volumes (0.15 mg/75 µL, 0.3 mg/75 µL, 0.3 mg/150 µL, and 0.6 mg/150 µL) and 20% Pluronic F-127 at varying volumes (75 µL and 150 µL). Each treatment group included six wounds. **iii.** Representative images of wounds for each treatment group at POD 13 histologically stained by Gomori's Trichrome (left) and immunohistologically stained for (top) cytokeratin 14. All scale bars represent 1 mm. **iv.** Quantification of wound re-epithelialization by representative cytokeratin 14 immunohistologically stained sections (top) and H&E stained sections (bottom) of wounds for each treatment group at POD 13 (n=6). **(B) Experimental design. i.** Experimental design illustrating allocation of different treatments to wound sites and animals. **ii.** Diagram of the planned wound sampling strategy for obtaining samples for various downstream analyses, such as histopathology and molecular analysis. This strategy ensured comparability and standardization of the wound sites used for each analysis. Data are presented as mean ± SD.H&E, hematoxylin & eosin; IHC, immunohistochemistry; POD, post-operation day; SD, standard deviation.

Major Resources Table

Animals (*in vivo* studies)

Species	Vendor or Source	Background Strain	Sex	Persistent ID / URL
Mouse	Animal Resources Centre, Australia	C57BL/6J	Male	
Pig	Oak Hill Genetics, Illinois, USA	Yorkshire	Female	https://www.oakhillgenetics.com/
Pig	Sinclair Research Center, Missouri, USA	Yucatan miniature	Male	https://www.altasciences.com/preclinical-research-services

Genetically Modified Animals

	Species	Vendor or Source	Background Strain	Other Information	Persistent ID / URL
**Parent - Male**	N/A	N/A	N/A	N/A	N/A
**Parent - Female**	N/A	N/A	N/A	N/A	N/A

N/A, not applicable

Antibodies

Target antigen	Vendor or Source	Catalog #	Working concentration	Lot # (preferred but not required)	Persistent ID / URL
Ly6G/C	Abcam	Ab2557	1:100	Clone NIMP-R14 Lot#GR3215393-1	https://www.abcam.com/en-us/products/primary-antibodies/neutrophil-antibody-nimp-r14-ab2557?srsltid=AfmBOopjjYOoDc2RFpEr_xwv_ROyUpgbhzfB_AwnFR-ArPH1oDkJsUwK
Alpha smooth muscle actin	Abcam	Ab5694	1:500	Lot#GR3356867-6	https://www.abcam.com/en-us/products/primary-antibodies/alpha-smooth-muscle-actin-antibody-ab5694?srsltid=AfmBOorEo2yMtE4fstI8KshA-4kY7B2dPqFHQ-RHTJvkvO3FGEcEysBA
CD68	Abcam	Ab125212	1:1000	GR3326063-10	https://www.abcam.com/en-us/products/primary-antibodies/cd68-antibody-ab125212
Cytokeratin-14 antibody	BioRad	MCA890T	1:3400	Clone LL002	https://www.bio-rad-antibodies.com/monoclonal/human-cytokeratin-14-antibody-ll002-mca890.html?f=purified&evCntryLang=DE-de&JSESSIONID_STERLING=adummyvalue

DNA/cDNA Clones

Clone Name	Sequence	Source / Repository	Persistent ID / URL
N/A	N/A	N/A	N/A

N/A, not applicable

Cultured Cells

Name	Vendor or Source	Sex (F, M, or unknown)	Persistent ID / URL
Murine macrophage cell line RAW264.7	ATCC	unknown	https://www.atcc.org/products/tib-71

Data & Code Availability

Description	Source / Repository	Persistent ID / URL
N/A	N/A	N/A

N/A, not applicable

Other

Description	Source / Repository	Persistent ID / URL
N/A	N/A	N/A

N/A, not applicable
